# Supplementary material for: Raman Spectroscopy as a Novel Method for the Characterization of Polydioxanone Medical Stents Biodegradation
Source: Materials (Basel). 2021 Sep 21;14(18):5462. doi: 10.3390/ma14185462 (PMC8467320; doi:10.3390/ma14185462)

Annex No. 6 to the document No. PŘL-72/19-DE
dated 20.04.2020 [signature]

č. 19-0859-61

Název:	**Termická analýza biodegradabilního polymeru**		
Přijetí vzorku:	05. 09.2019		
Vypracoval:	Z. Křenková	Zkontroloval:	T. Čermák
Zadavatel:	ELLA-CS s.r.o., Milady Horákove 504/45, 500 06 Hradec Králové		

Výsledky:

Byla provedena termická analýza biodegradabilního polymeru:
D6-02-16, Lot S19001245-01, CETA č. 288/19 (nedegradovaný)

Výsledky diferenciální skenovací kalorimetrie (DSC) jsou uvedeny v tabulce č. 1 (teploty tání a krystalizace, entalpie tání a studené krystalizace, skelný přechod) a v tabulce č. 2 (krystalinita).
Měření DSC jsou zobrazena na obrázcích č. 1 – 3.
Měření termogravimetrické analýzy (TGA) jsou zobrazena na obrázcích č. 4 a 5.

Poznámky:

Analýza byla provedena v souladu s normami:

- ČSN EN ISO 11357-1 Plasty – Diferenciální snímací kalorimetrie (DSC) – Část 1: Základní principy
- ČSN EN ISO 11357-2 Plasty - Diferenciální snímací kalorimetrie (DSC) – Část 2: Stanovení teploty a výšky skoku skelného přechodu
- ČSN EN ISO 11357-3 Plasty - Diferenciální snímací kalorimetrie (DSC) – Část 3: Stanovení teploty a entalpie tání a krystalizace
- ČSN EN ISO 11358-1 Plasty – Termogravimetrie (TG) polymerů – Část 1: Obecné principy

Ing. Michal Bartoš
Vedoucí úseku ANALYTIKA

Signature valid
Digitálně podepsáno
Jméno: Ing. Michal Bartoš
Datum: 16.10.2019 16:47:33

Výzkumný ústav organických syntéz a.s.
č.p. 296,
533 54 Rybitví
CZECH REPUBLIC

Tel: +420 466 822 501
e-mail:analytika@vuos.com
homepage:www.vuos.com

Registrováno: Krajský soud Hradec Králové
oddíl B, vložka 1033
DIČ: CZ60108975

Analyzovaný vzorek:

D6-02-16, Lot S19001245-01, CETA č. 288/19 (nedegradovaný)
Materiál polydioxanon (PDO).
(Skladování v exsikátoru při laboratorní teplotě)

Diferenční skenovací kalorimetrie

Přístrojové vybavení:

DSC823[e] Mettler Toledo

- Měřící teplotní rozsah: 203 – 973 K (-70 to 700 °C)
- Rozlišení: přibližně 0.07 µW
- Měřící kelímek: hliníkový kelímek (100µl), zalisovaný víčkem s otvorem vytvořeným speciálním bodlem
- Referenční kelímek: prázdný hliníkový kelímek (100µl), zalisovaný víčkem s otvorem vytvořeným speciálním bodlem

S ohledem na požadavky hodnocení krystalinity byly vzorky navažovány na mikrovahách (součást zařízení TGA/DSC1 Mettler Toledo).

Metoda:

Krok		Teplotní rozsah [°C]	Čas isotermy [min]/ teplotní gradient [°C/min]
1.	isotermický krok	25	5 min
2.	dynamický krok – 1.ohřev	25 – 150	5 °C/min
3.	isotermický krok	150	5 min
4.	dynamický krok – chlazení	150 - -30	5 °C/min
5.	isotermický krok	-30	5 min
6.	dynamický krok – 2.ohřev	-30 - 150	5 °C/min

Termogravimetrická analýza

Přístrojové vybavení:

TGA/DSC1 Mettler Toledo

- Teplotní rozsah: 298 – 1373 K (25 – 1100 °C)
- Citlivost: 0.5 mW
- Teplotní rozlišení: 0.005 K
- Měřící kelímek: hliníkový kelímek (100µl), zalisovaný víčkem s otvorem vytvořeným speciálním bodlem

Metoda:

Teplotní rozsah [°C] 25 – 400 °C (1.měření) a 25 – 300 °C (2. měření)
teplotní gradient [°C/min] 5 °C/min
Průtok inertního plynu (dusík) byl nastaven na cca 80 ml/min.

Hodnocení a závěr:

Diferenční skenovací kalorimetrie

Tabulka č. 1: Termická charakterizace vzorku D6-02-16, Lot S19001245-01, CETA č. 288/19

Stanovení č.	Teplota tání			Entalpie tání	Teplota krystalizace		Skelný přechod	Entalpie studené krystalizace	Entalpie studené krystalizace (premelting)	Entalpie tání
	**T_m (onset) [°C]**	**Peak 1 [°C]**	**Peak 2 [°C]**	**ΔH_f [J/g]**	**T_c (onset) [°C]**	**Peak [°C]**	**Tg [°C]**	**ΔH_c [J/g]**	**ΔH_c [J/g]**	**ΔH_f [J/g]**
	**1.ohřev**			**1.ohřev**	**chlazení**		**2.ohřev**	**2. ohřev**	**2. ohřev**	**2.ohřev**
**1**	94,12	96,52	109,08	63,37	67,69	49,20	-10,07	24,02	3,64	47,98
**2**	93,77	97,46	105,19	69,02	67,49	51,29	-10,24	25,07	4,08	49,90
**3**	93,99	98,18	108,42	69,11	67,03	50,79	-10,32	26,31	4,26	51,11
**Průměr**	**94,0**	**97,4**	**107,6**	**67,2**	**67,4**	**50,4**	**-10,2**	**25,1**	**4,0**	**49,7**
SD	0,2	0,8	2,1	3,3	0,3	1,1	0,1	1,1	0,3	1,6

Onset teplota ...... extrapolovaná počáteční teplota (průsečík extrapolované základní čáry tepelného toku s tečnou v inflexním bodu píku)

Výpočty:

$$Xc = \frac{\Delta H_f - \Delta H_c}{\Delta H_{f\,100\%}} \times 100$$

X_c..............................Krystalinita; procentuální podíl krystalické fáze polymeru [%]

ΔH_f..........................Entalpie tání; teplo pohlcené v průběhu tání vzorku [J/g]

ΔH_c ....Entalpie studené krystalizace; teplo uvolněné v průběhu studené krystalizace vzorku [J/g]

$\Delta H_{f\,100\%}$..................Teoretická hodnota entalpie tání 100% krystalického polymeru PDO [J/g]; 141,18 J/g (zdroj: Thermal properties and non-isothermal crystallization behavior of biodegradable poly(p-dioxanone)/poly(vinyl alcohol) blends; DOI: 10.1002/pi.1904)

Krystalinita polymeru (Xc) vyhodnocená během 1. ohřevu charakterizuje polymer ("as received") s jeho teplotní a mechanickou historií (vliv výrobního postupu, skladování atd.). Krystalinita vyhodnocená během 2. ohřevu charakterizuje polymer po vymazání jeho teplotní i mechanické historie a slouží především ke srovnávání polymerních materiálů vzájemně.

Tabulka č. 2: Krystalinita vzorku D6-02-16, Lot S19001245-01, CETA č. 288/19 vyhodnocená během 1. a 2. ohřevu

Stanovení č.	Krystalinita, X_c [%]	
	1.ohřev	2. ohřev
**1**	44,9	14,4
**2**	48,9	14,7
**3**	49,0	14,5
**Průměr**	**47,6**	**14,5**
SD	2,3	0,2

Obrázek č. 1: DSC vzorku D6-02-16, Lot S19001245-01, CETA č. 288/19 (1. měření)

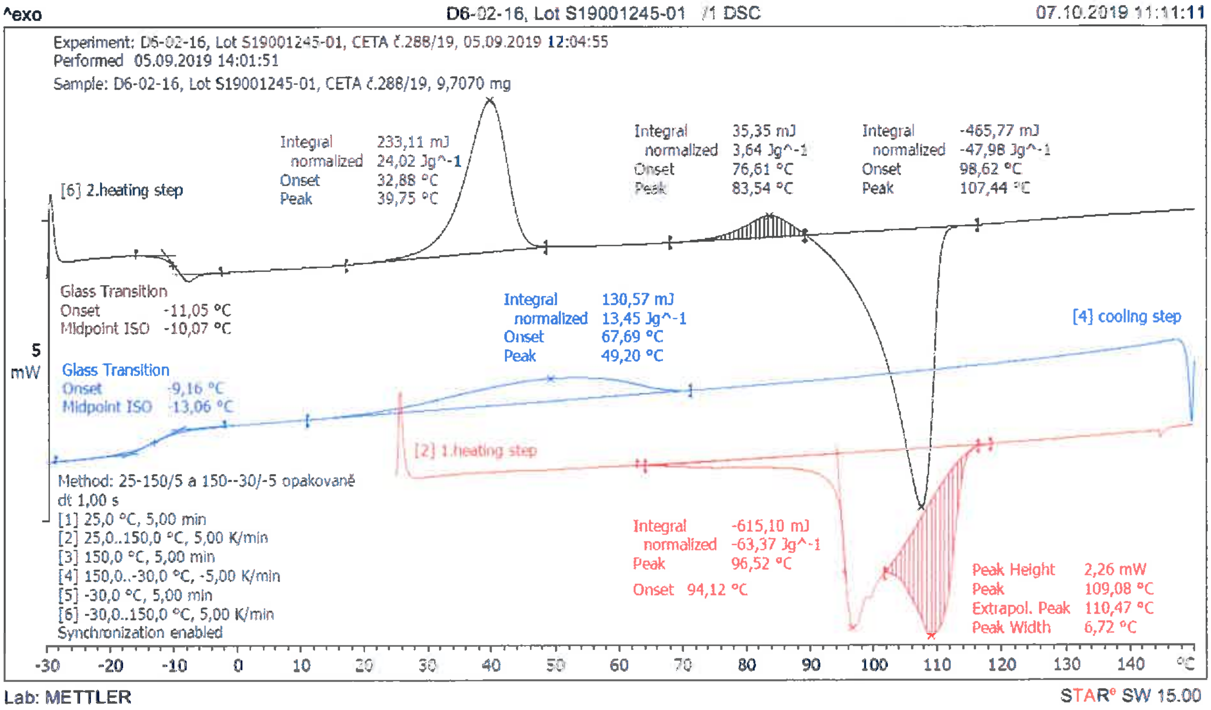

Obrázek č. 2: DSC vzorku D6-02-16, Lot S19001245-01, CETA č. 288/19 (2. měření)

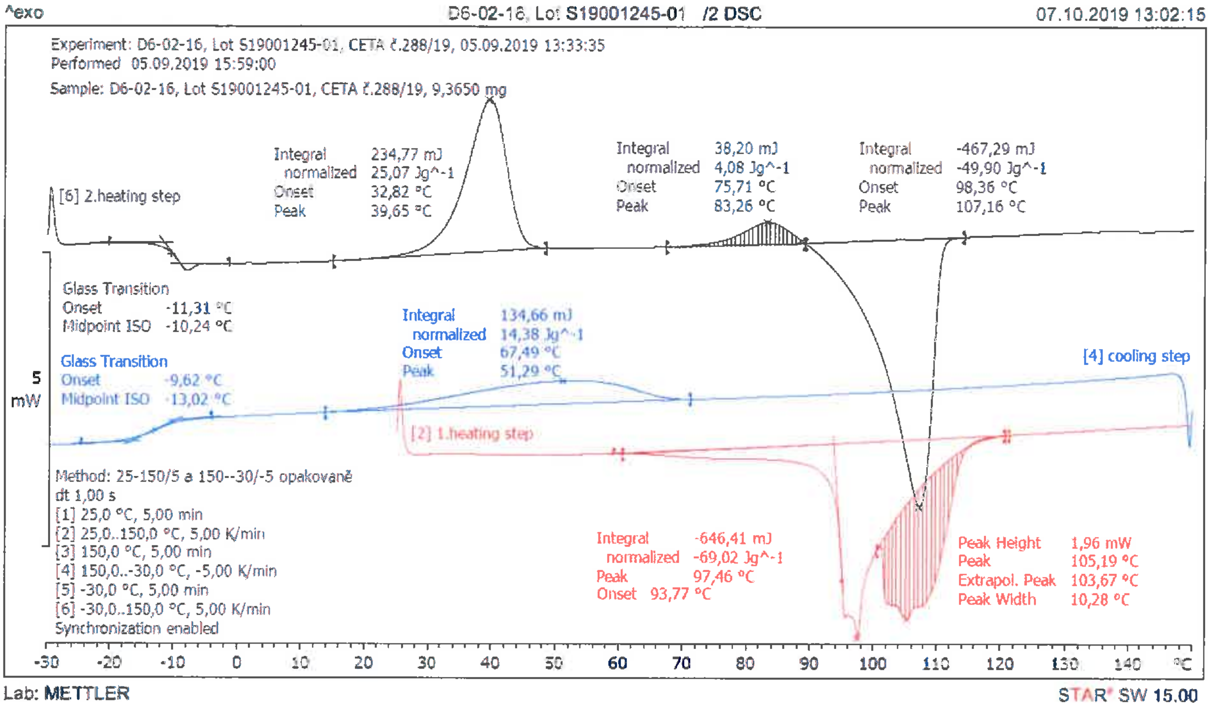

Obrázek č. 3: DSC vzorku D6-02-16, Lot S19001245-01, CETA č. 288/19 (3. měření)

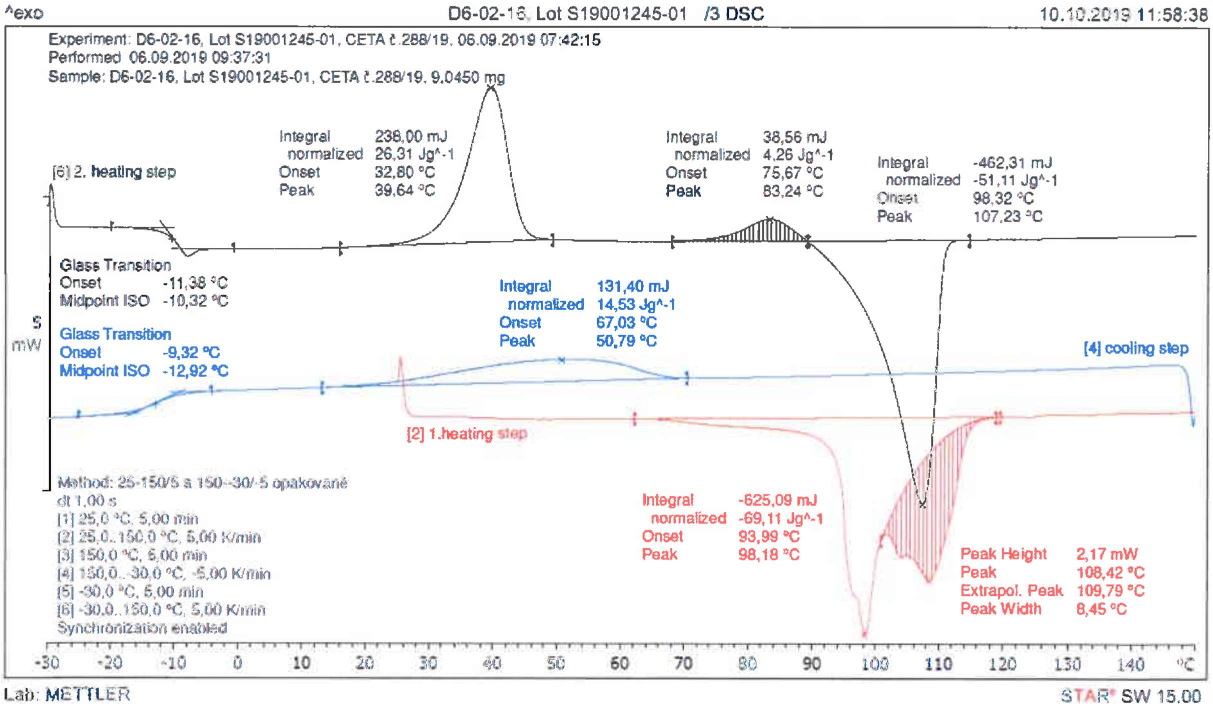

Termogravimetrická analýza

Na obrázcích č. 4 a 5 jsou zobrazena měření TGA u vzorku D6-02-16, Lot S19001245-01, CETA č. 288/19. Na hmotnostní křivce (černá křivka) nebyl zaznamenán žádný hmotnostní úbytek od 25 °C do 220 °C. Od cca 220 °C začíná termický rozklad polymeru. Na křivce tepelného toku (modrá křivka) byl zaznamenán zdvojený endotermický pík charakterizovaný onset teplotou 94,70 °C (1. měření) a 92,78 °C (2. měření), odpovídající tání polymeru. Druhý endotermický pík odpovídá termickému rozkladu polymeru a je charakterizovaný onset teplotou 270,36 °C (1. měření) a 264,67 °C (2. měření).

Obrázek č. 4: TGA vzorku D6-02-16, Lot S19001245-01, CETA č. 288/19 (1. měření)

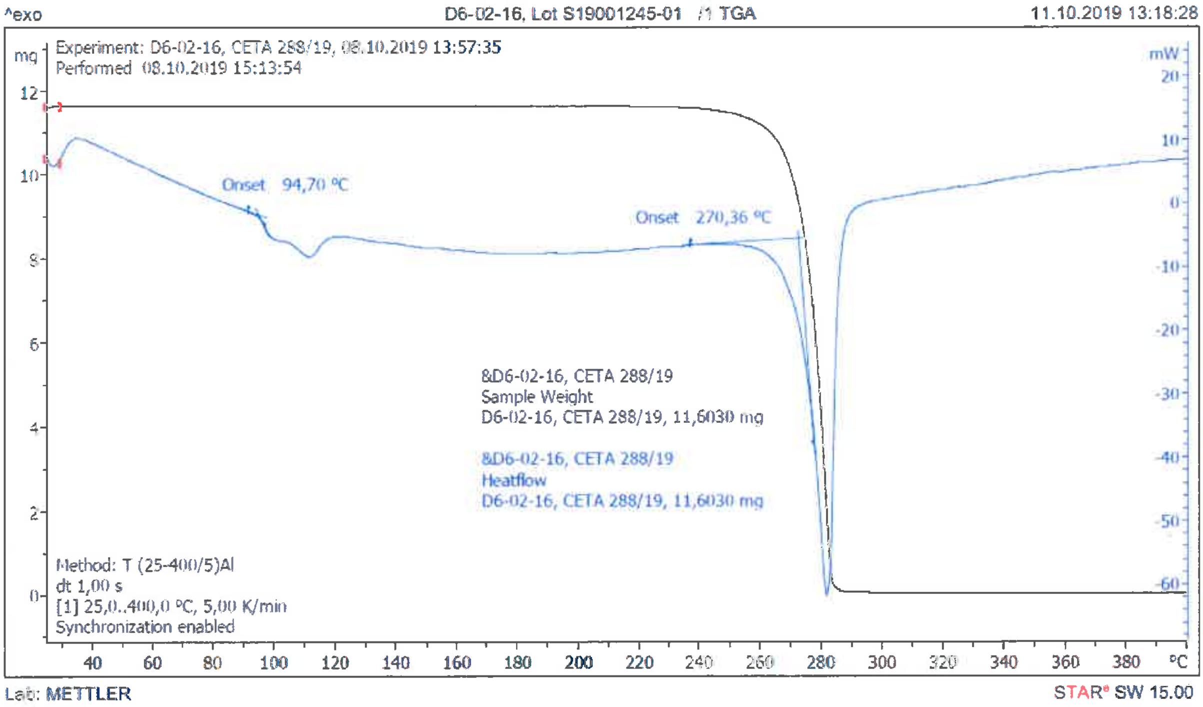

Obrázek č. 5: TGA vzorku D6-02-16, Lot S19001245-01, CETA č. 288/19 (2. měření)

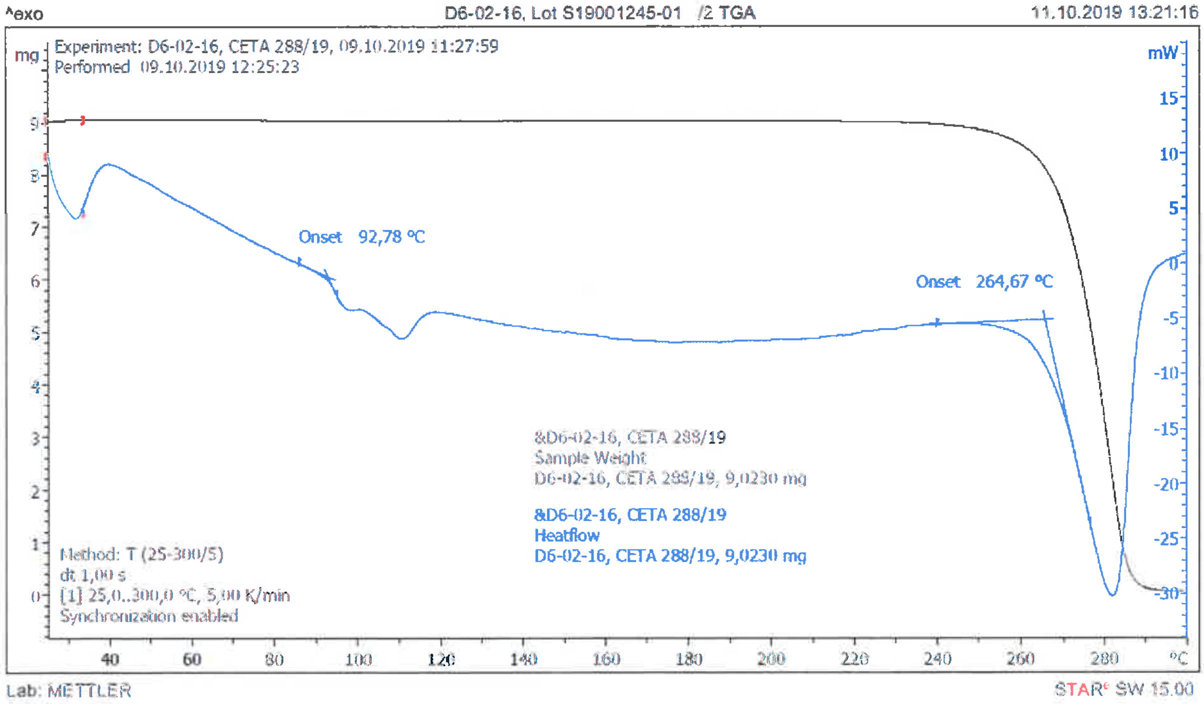

č. 19-0860-61

Název:	**Termická analýza biodegradabilního polymeru**		
Přijetí vzorku:	05. 09. 2019		
Vypracoval:	Z. Křenková	Zkontroloval:	T. Čermák
Zadavatel:	ELLA-CS s.r.o., Milady Horákove 504/45, 500 06 Hradec Králové		

Výsledky:

Byla provedena termická analýza biodegradabilního polymeru:
D6-02-04, Lot S19001245-01, CETA č. 289/19 (4 týdny degradovaný)

Výsledky diferenciální skenovací kalorimetrie (DSC) jsou uvedeny v tabulce č. 1 (teploty tání a krystalizace, entalpie tání a studené krystalizace, skelný přechod) a v tabulce č. 2 (krystalinita). Měření DSC jsou zobrazena na obrázcích č. 1 – 3.
Měření termogravimetrické analýzy (TGA) jsou zobrazena na obrázcích č. 4 a 5.

Poznámky:

Analýza byla provedena v souladu s normami:

- ČSN EN ISO 11357-1 Plasty – Diferenciální snímací kalorimetrie (DSC) – Část 1: Základní principy
- ČSN EN ISO 11357-2 Plasty - Diferenciální snímací kalorimetrie (DSC) – Část 2: Stanovení teploty a výšky skoku skelného přechodu
- ČSN EN ISO 11357-3 Plasty - Diferenciální snímací kalorimetrie (DSC) – Část 3: Stanovení teploty a entalpie tání a krystalizace
- ČSN EN ISO 11358-1 Plasty – Termogravimetrie (TG) polymerů – Část 1: Obecné principy

Ing. Michal Bartoš
Vedoucí úseku ANALYTIKA

Signature valid
Digitálně podepsáno
Jméno: Ing. Michal Bartoš
Datum: 16.10.2019 16:47:54

Analyzovaný vzorek:

D6-02-04, Lot S19001245-01, CETA č. 289/19 (4 týdny degradovaný)
Materiál polydioxanon (PDO).
(Skladování v exsikátoru při laboratorní teplotě)

Diferenční skenovací kalorimetrie

Přístrojové vybavení:

DSC823^e Mettler Toledo
- Měřící teplotní rozsah: 203 – 973 K (-70 to 700 °C)
- Rozlišení: přibližně 0.07 µW
- Měřící kelímek: hliníkový kelímek (100µl), zalisovaný víčkem s otvorem vytvořeným speciálním bodlem
- Referenční kelímek: prázdný hliníkový kelímek (100µl), zalisovaný víčkem s otvorem vytvořeným speciálním bodlem

S ohledem na požadavky hodnocení krystalinity byly vzorky navažovány na mikrovahách (součást zařízení TGA/DSC1 Mettler Toledo).

Metoda:

Krok		Teplotní rozsah [°C]	Čas isotermy [min]/ teplotní gradient [°C/min]
1.	isotermický krok	25	5 min
2.	dynamický krok – 1.ohřev	25 – 150	5 °C/min
3.	isotermický krok	150	5 min
4.	dynamický krok – chlazení	150 - -30	5 °C/min
5.	isotermický krok	-30	5 min
6.	dynamický krok – 2.ohřev	-30 - 150	5 °C/min

Termogravimetrická analýza

Přístrojové vybavení:

TGA/DSC1 Mettler Toledo
- Teplotní rozsah: 298 – 1373 K (25 – 1100 °C)
- Citlivost: 0.5 mW
- Teplotní rozlišení: 0.005 K
- Měřící kelímek: hliníkový kelímek (100µl), zalisovaný víčkem s otvorem vytvořeným speciálním bodlem

Metoda:

Teplotní rozsah [°C] 25 – 400 °C (1.měření) a 25 – 300 °C (2. měření)
teplotní gradient [°C/min] 5 °C/min
Průtok inertního plynu (dusík) byl nastaven na cca 80 ml/min.

Hodnocení a závěr:

Diferenční skenovací kalorimetrie

Tabulka č. 1: Termická charakterizace vzorku D6-02-04, Lot S19001245-01, CETA č. 289/19 (4 týdny degradovaný)

Stanovení č.	Teplota tání			Entalpie tání	Teplota krystalizace		Skelný přechod	Entalpie studené krystalizace	Entalpie studené krystalizace (premelting)	Entalpie tání
	**T_m (onset) [°C]**	**Peak 1 [°C]**	**Peak 2 [°C]**	**ΔH$_f$ [J/g]**	**T_c (onset) [°C]**	**Peak [°C]**	**Tg [°C]**	**ΔH$_c$ [J/g]**	**ΔH$_c$ [J/g]**	**ΔH$_f$ [J/g]**
	**1.ohřev**			**1.ohřev**	**chlazení**		**2.ohřev**	**2. ohřev**	**2. ohřev**	**2.ohřev**
**1**	95,92	101,67	109,98	76,18	64,36	47,30	-10,31	23,92	6,17	58,60
**2**	96,09	100,68	109,51	76,94	64,98	48,97	-10,56	23,40	6,05	59,13
**3**	95,84	100,91	110,17	79,07	64,78	46,72	-10,06	22,74	6,48	59,78
**Průměr**	**96,0**	**101,1**	**109,9**	**77,4**	**64,7**	**47,7**	**-10,3**	**23,4**	**6,2**	**59,2**
SD	0,1	0,5	0,3	1,5	0,3	1,2	0,3	0,6	0,2	0,6

Onset teplota ...... extrapolovaná počáteční teplota (průsečík extrapolované základní čáry tepelného toku **s** tečnou v inflexním bodu píku)

Výpočty:

$$Xc = \frac{\Delta H_f - \Delta H_c}{\Delta H_{f\ 100\%}} \times 100$$

X_c..............................Krystalinita; procentuální podíl krystalické fáze polymeru [%]
ΔH$_f$...........................Entalpie tání; teplo pohlcené v průběhu tání vzorku [J/g]
ΔH$_c$..........................Entalpie studené krystalizace; teplo uvolněné v průběhu studené krystalizace vzorku [J/g]
ΔH$_{f\ 100\%}$..................Teoretická hodnota entalpie tání 100% krystalického polymeru PDO [J/g]; 141,18 J/g (zdroj: Thermal properties and non-isothermal crystallization behavior of biodegradable poly(p-dioxanone)/poly(vinyl alcohol) blends; DOI: 10.1002/pi.1904)

Krystalinita polymeru (Xc) vyhodnocená během 1. ohřevu charakterizuje polymer ("as received") s jeho teplotní a mechanickou historií (vliv výrobního postupu, skladování atd.). Krystalinita vyhodnocená během 2. ohřevu charakterizuje polymer po vymazání jeho teplotní i mechanické historie a slouží především ke srovnávání polymerních materiálů vzájemně.

Tabulka č. 2: Krystalinita vzorku D6-02-04, Lot S19001245-01, CETA č. 289/19 (4 týdny degradovaný) vyhodnocená během 1. a 2. ohřevu

**Stanovení č.**	**Krystalinita, X_c [%]**	
	**1.ohřev**	**2. ohřev**
**1**	53,96	20,19
**2**	54,50	21,02
**3**	56,01	21,65
**Průměr**	**54,8**	**21,0**
SD	1,1	0,7

Obrázek č. 1: DSC vzorku D6-02-04, Lot S19001245-01, CETA č. 289/19 (4 týdny degradovaný) (1. měření)

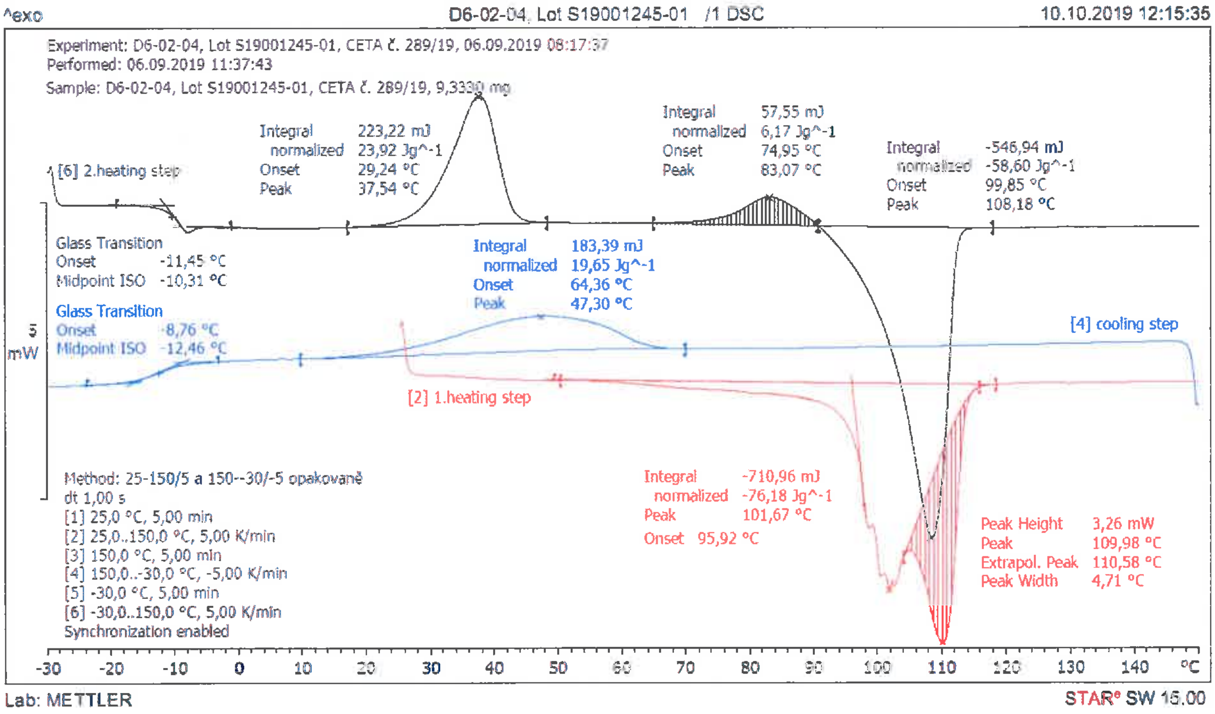

Obrázek č. 2: DSC vzorku D6-02-04, Lot S19001245-01, CETA č. 289/19 (4 týdny degradovaný) (2. měření)

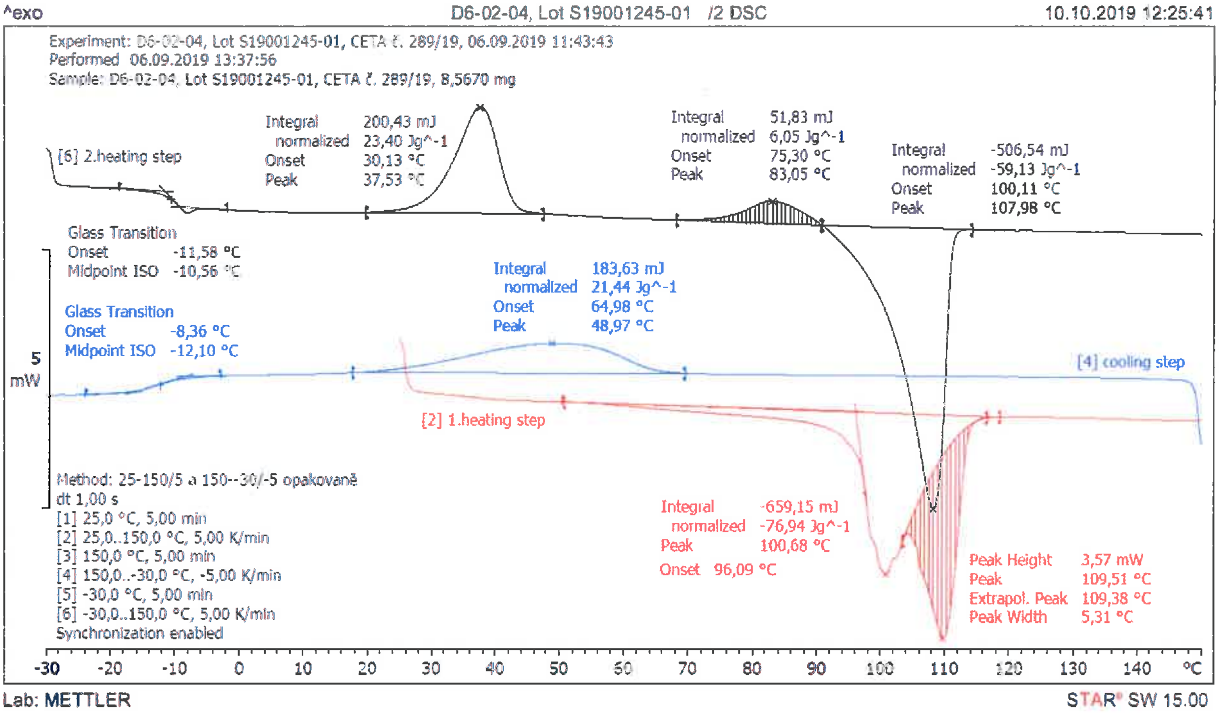

Obrázek č. 3: DSC vzorku D6-02-04, Lot S19001245-01, CETA č. 289/19 (4 týdny degradovaný) (3. měření)

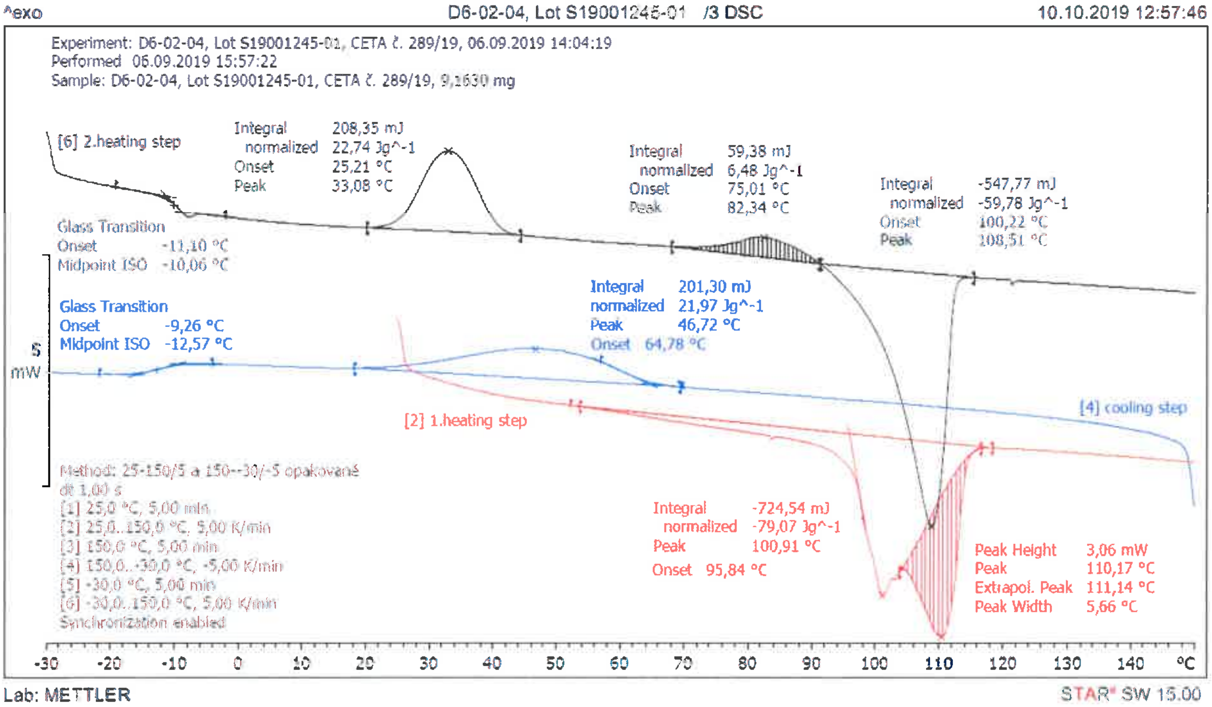

Termogravimetrická analýza

Na obrázcích č. 4 a 5 jsou zobrazena měření TGA u vzorku D6-02-04, Lot S19001245-01, CETA č. 289/19 (4 týdny degradovaný). Na hmotnostní křivce (černá křivka) nebyl zaznamenán žádný hmotnostní úbytek od 25 °C do 220 °C. Od cca 220 °C začíná termický rozklad polymeru. Na křivce tepelného toku (modrá křivka) byl zaznamenán zdvojený endotermický pík charakterizovaný onset teplotou 96,35 °C (1. měření) a 95,88 °C (2. měření), odpovídající tání polymeru. Druhý endotermický pík odpovídá termickému rozkladu polymeru a je charakterizovaný onset teplotou 270,27 °C (1. měření) a 270,83 °C (2. měření).

Obrázek č. 4: TGA vzorku D6-02-04, Lot S19001245-01, CETA č. 289/19 (4 týdny degradovaný) (1. měření)

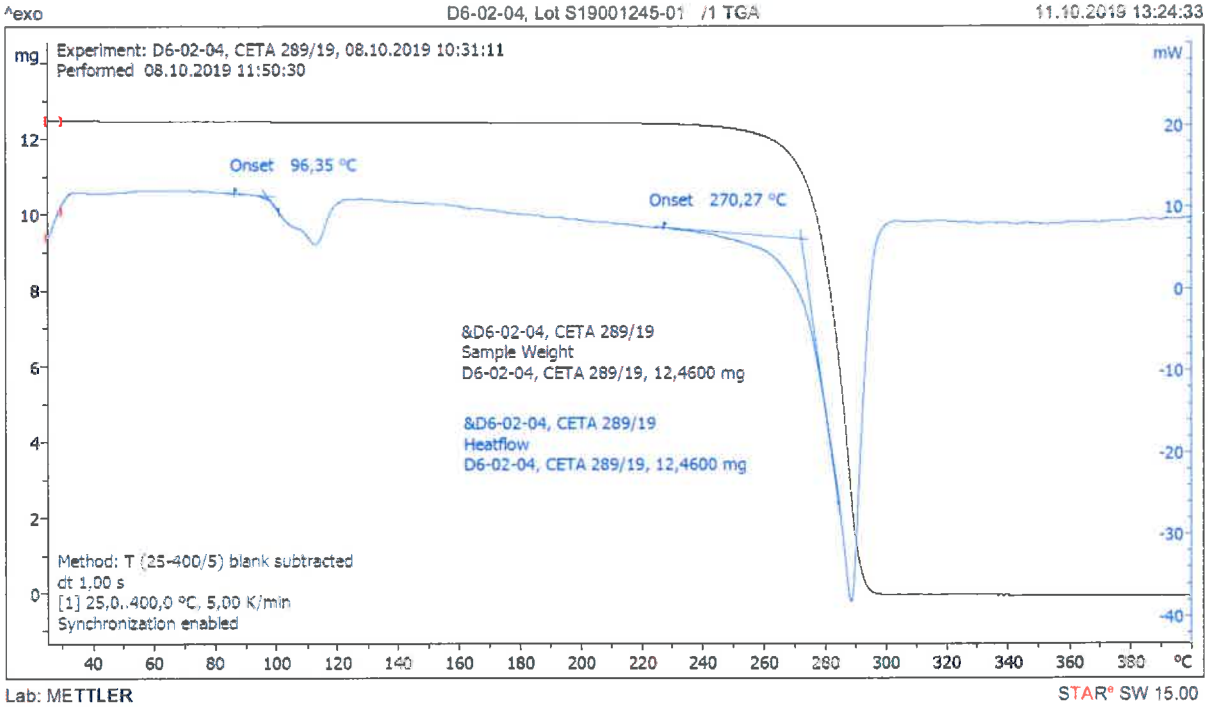

Obrázek č. 5: TGA vzorku D6-02-04, Lot S19001245-01, CETA č. 289/19 (4 týdny degradovaný) (2. měření)

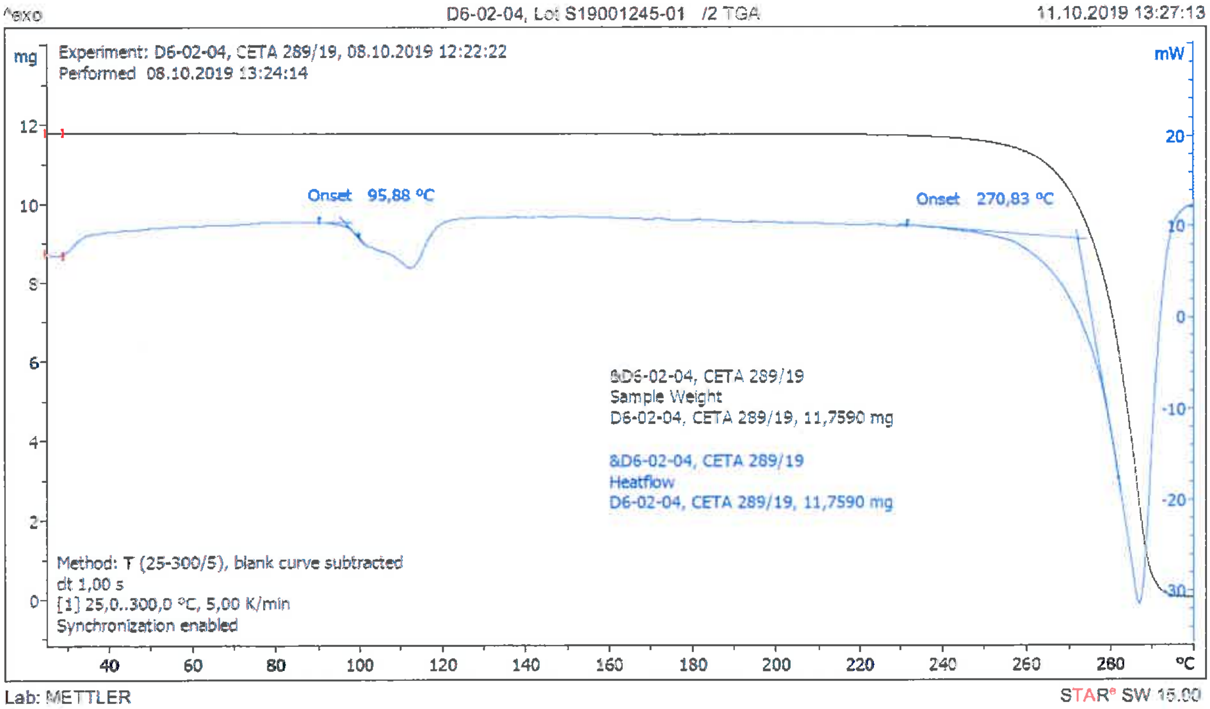

č. 19-1045-61

Název:	**Termická analýza biodegradabilního polymeru**		
Přijetí vzorku:	02. 10. 2019		
Vypracoval:	Z. Křenková	Zkontroloval:	T. Čermák
Zadavatel:	ELLA-CS s.r.o., Milady Horákové 504/45, 500 06 Hradec Králové		

Výsledky:

Byla provedena termická analýza biodegradabilního polymeru:
D6-02-07, Lot S19001245-01, CETA č. 315/19 (8 týdnů degradovaný)

Výsledky diferenciální skenovací kalorimetrie (DSC) jsou uvedeny v tabulce č. 1 (teploty tání a krystalizace, entalpie tání a studené krystalizace, skelný přechod) a v tabulce č. 2 (krystalinita).
Měření DSC jsou zobrazena na obrázcích č. 1 – 3.
Měření termogravimetrické analýzy (TGA) jsou zobrazena na obrázcích č. 4 a 5.

Poznámky:

Analýza byla provedena v souladu s normami:
- ČSN EN ISO 11357-1 Plasty – Diferenciální snímací kalorimetrie (DSC) – Část 1: Základní principy
- ČSN EN ISO 11357-2 Plasty - Diferenciální snímací kalorimetrie (DSC) – Část 2: Stanovení teploty a výšky skoku skelného přechodu
- ČSN EN ISO 11357-3 Plasty - Diferenciální snímací kalorimetrie (DSC) – Část 3: Stanovení teploty a entalpie tání a krystalizace
- ČSN EN ISO 11358-1 Plasty – Termogravimetrie (TG) polymerů – Část 1: Obecné principy

Ing. Michal Bartoš
Vedoucí úseku ANALYTIKA

Signature valid
Digitálně podepsáno
Jméno: Ing. Michal Bartoš
Datum: 05.12.2019
14:04:49

Analyzovaný vzorek:

Název: D6-02-07, Lot S19001245-01, CETA č. 315/19 (8 týdnů degradovaný)
Materiál: Polydioxanon (PDO).
Skladování: Exsikátor, laboratorní teplota

Diferenční skenovací kalorimetrie

Přístrojové vybavení:

DSC823^e Mettler Toledo

- Měřící teplotní rozsah: 203 – 973 K (-70 to 700 °C)
- Rozlišení: přibližně 0.07 µW
- Měřící kelímek: hliníkový kelímek (100µl), zalisovaný víčkem s otvorem vytvořeným speciálním bodlem
- Referenční kelímek: prázdný hliníkový kelímek (100µl), zalisovaný víčkem s otvorem vytvořeným speciálním bodlem

S ohledem na požadavky hodnocení krystalinity byly vzorky navažovány na mikrovahách (součást zařízení TGA/DSC1 Mettler Toledo).

Metoda:

Krok		Teplotní rozsah [°C]	Čas isotermy [min]/ teplotní gradient [°C/min]
1.	isotermický krok	25	5 min
2.	dynamický krok – 1.ohřev	25 – 150	5 °C/min
3.	isotermický krok	150	5 min
4.	dynamický krok – chlazení	150 - -30	5 °C/min
5.	isotermický krok	-30	5 min
6.	dynamický krok – 2.ohřev	-30 - 150	5 °C/min

Termogravimetrická analýza

Přístrojové vybavení:

TGA/DSC1 Mettler Toledo

- Teplotní rozsah: 298 – 1373 K (25 – 1100 °C)
- Citlivost: 0.5 mW
- Teplotní rozlišení: 0.005 K
- Měřící kelímek: hliníkový kelímek (100µl), zalisovaný víčkem s otvorem vytvořeným speciálním bodlem

Metoda:

Krok		Teplotní rozsah [°C]	Teplotní gradient [°C/min]
1.	dynamický krok	25 – 400	5

Průtok inertního plynu (dusík) byl nastaven na cca 80 ml/min.

Hodnocení a závěr:

Diferenční skenovací kalorimetrie

Tabulka č. 1: Termická charakterizace vzorku D6-02-07, Lot S19001245-01, CETA č. 315/19 (8 týdnů degradovaný)

Stanovení č.	Teplota tání			Entalpie tání	Teplota krystalizace		Skelný přechod	Entalpie studené krystalizace	Entalpie studené krystalizace (premelting)	Entalpie tání
	**T_m (onset) [°C]**	**Peak 1 [°C]**	**Peak 2 [°C]**	**ΔH_f [J/g]**	**T_c (onset) [°C]**	**Peak [°C]**	**Tg [°C]**	**ΔH_c [J/g]**	**ΔH_c [J/g]**	**ΔH_f [J/g]**
	**1.ohřev**			**1.ohřev**	**chlazení**		**2.ohřev**	**2. ohřev**	**2. ohřev**	**2.ohřev**
**1**	100,87	-	111,15	86,83	69,94	50,49	-12,64	12,10	9,33	72,74
**2**	101,29	-	111,22	86,10	69,62	50,74	-12,52	12,50	7,02	75,32
**3**	101,63	-	111,29	84,95	68,75	52,40	-12,55	10,30	8,52	71,93
**Průměr**	**101,3**	**-**	**111,2**	**86,0**	**69,4**	**51,2**	**-12,6**	**11,6**	**8,3**	**73,3**
SD	0,4	-	0,1	0,9	0,6	1,0	0,1	1,2	1,2	1,8

Onset teplota ...... extrapolovaná počáteční teplota (průsečík extrapolované základní čáry tepelného toku s tečnou v inflexním bodu píku)

Výpočty:

$$Xc = \frac{\Delta H_f - \Delta H_c}{\Delta H_{f\ 100\%}} \times 100$$

X_c..............................Krystalinita; procentuální podíl krystalické fáze polymeru [%]
ΔH_f..........................Entalpie tání; teplo pohlcené v průběhu tání vzorku [J/g]
ΔH_c ....Entalpie studené krystalizace; teplo uvolněné v průběhu studené krystalizace vzorku [J/g]
$\Delta H_{f\ 100\%}$..................Teoretická hodnota entalpie tání 100% krystalického polymeru PDO [J/g]; 141,18 J/g (zdroj: Thermal properties and non-isothermal crystallization behavior of biodegradable poly(p-dioxanone)/poly(vinyl alcohol) blends; DOI: 10.1002/pi.1904)

Krystalinita polymeru (Xc) vyhodnocená během 1. ohřevu charakterizuje polymer ("as received") s jeho teplotní a mechanickou historií (vliv výrobního postupu, skladování atd.). Krystalinita vyhodnocená během 2. ohřevu charakterizuje polymer po vymazání jeho teplotní i mechanické historie a slouží především ke srovnávání polymerních materiálů vzájemně.

Tabulka č. 2: Krystalinita vzorku D6-02-07, Lot S19001245-01, CETA č. 315/19 (8 týdnů degradovaný) vyhodnocená během 1. a 2. ohřevu

**Stanovení č.**	**Krystalinita, X_c [%]**	
	**1.ohřev**	**2. ohřev**
**1**	61,50	36,34
**2**	60,99	39,52
**3**	60,17	37,62
**Průměr**	**60,9**	**37,8**
SD	0,7	1,6

Obrázek č. 1: DSC vzorku D6-02-07, Lot S19001245-01, CETA č. 315/19 (8 týdnů degradovaný) (1. měření)

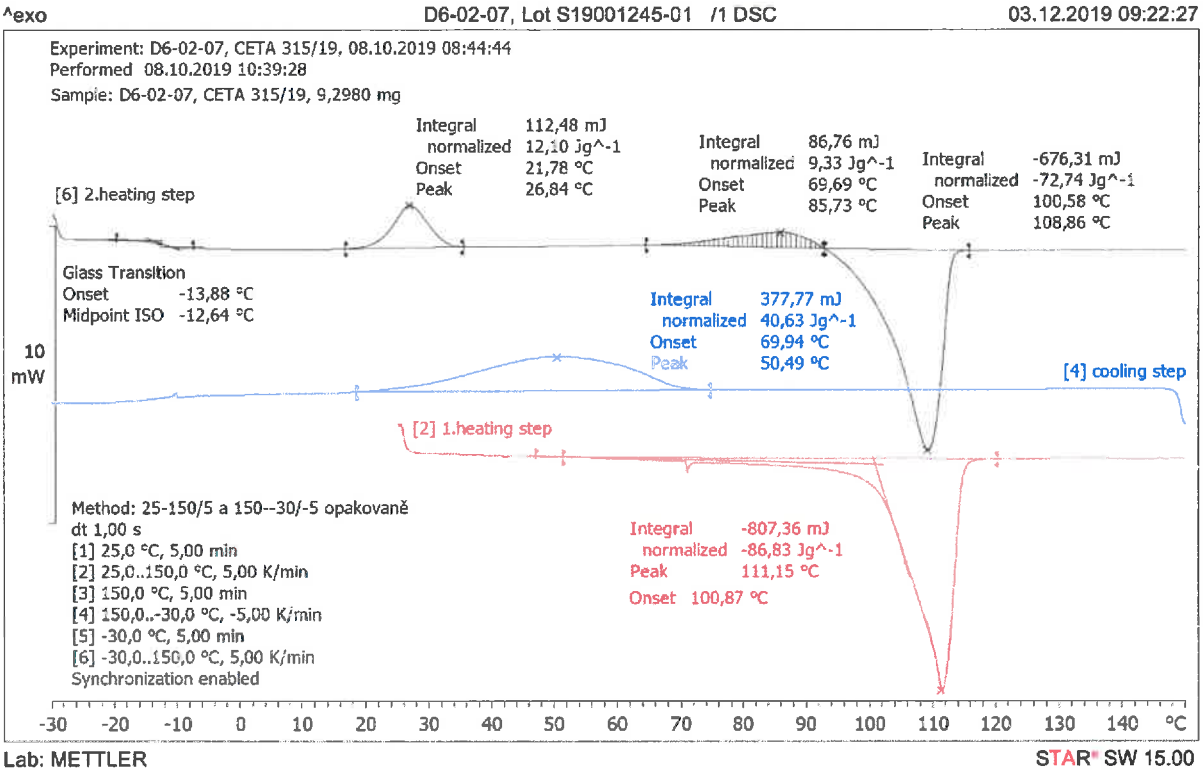

Obrázek č. 2: DSC vzorku D6-02-07, Lot S19001245-01, CETA č. 315/19 (8 týdnů degradovaný) (2. měření)

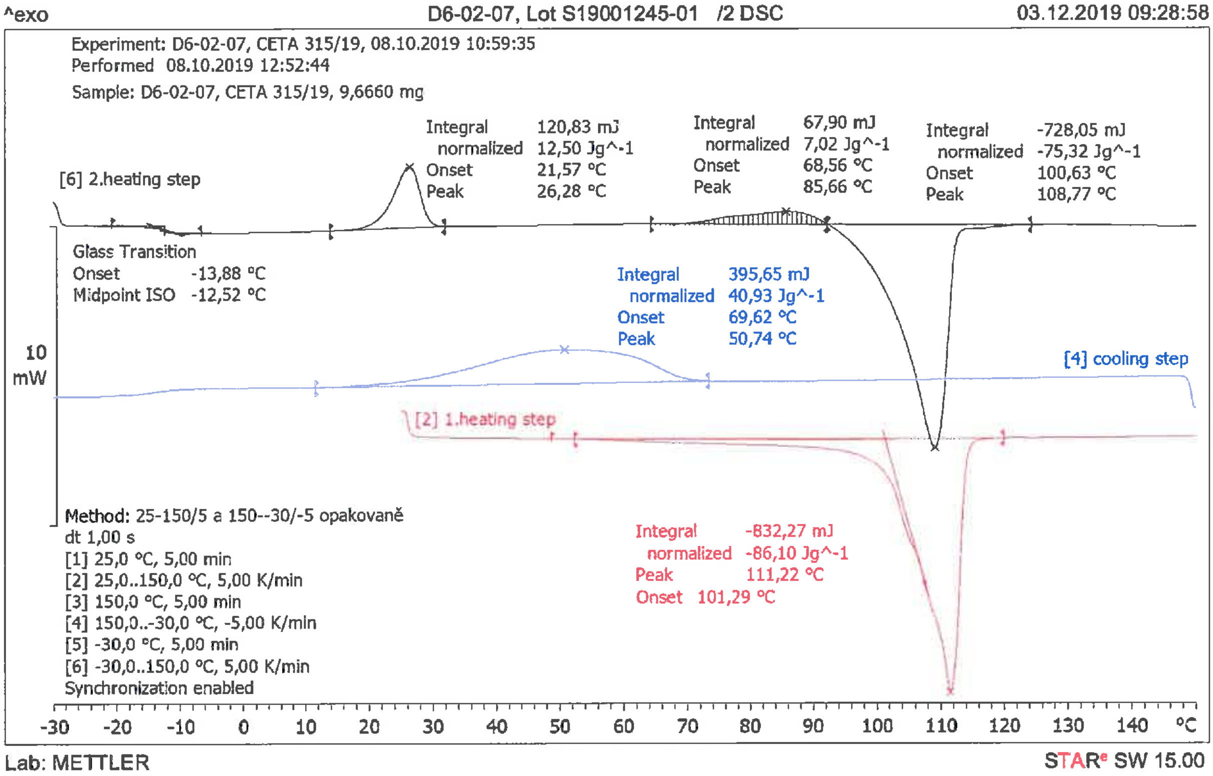

Obrázek č. 3: DSC vzorku D6-02-07, Lot S19001245-01, CETA č. 315/19 (8 týdnů degradovaný) (3. měření)

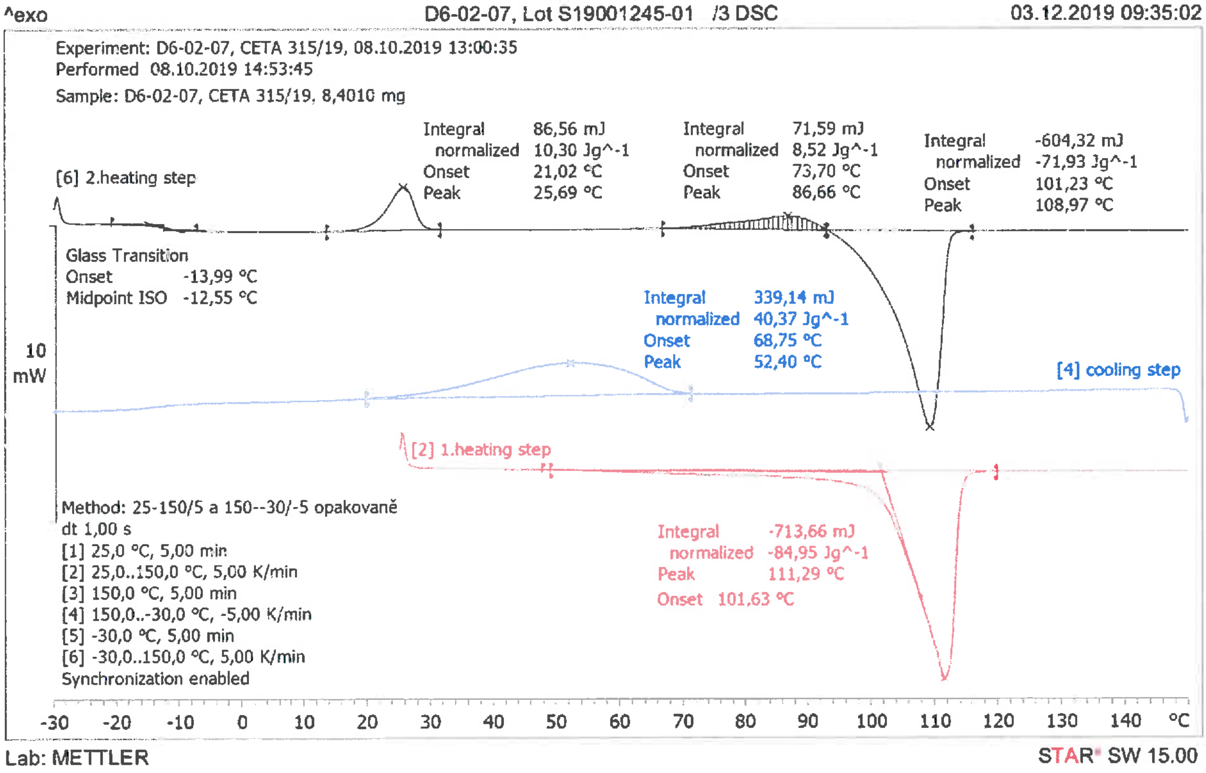

Termogravimetrická analýza

Na obrázcích č. 4 a 5 jsou zobrazena měření TGA u vzorku D6-02-07, Lot S19001245-01, CETA č. 315/19 (8 týdnů degradovaný). Na hmotnostní křivce (černá křivka) nebyl zaznamenán žádný hmotnostní úbytek od 25 °C do 180 °C. Od cca 180 °C začíná termický rozklad polymeru. Na křivce tepelného toku (modrá křivka) byl zaznamenán endotermický pík charakterizovaný teplotou píku 112,24 (1. měření) a 112,18 °C (2. měření), odpovídající tání polymeru. Druhý endotermický pík odpovídá termickému rozkladu polymeru a je charakterizovaný onset teplotou 254,21 °C (1. měření) a 262,23 °C (2. měření).

Obrázek č. 4: TGA vzorku D6-02-07, Lot S19001245-01, CETA č. 315/19 (8 týdnů degradovaný) (1. měření)

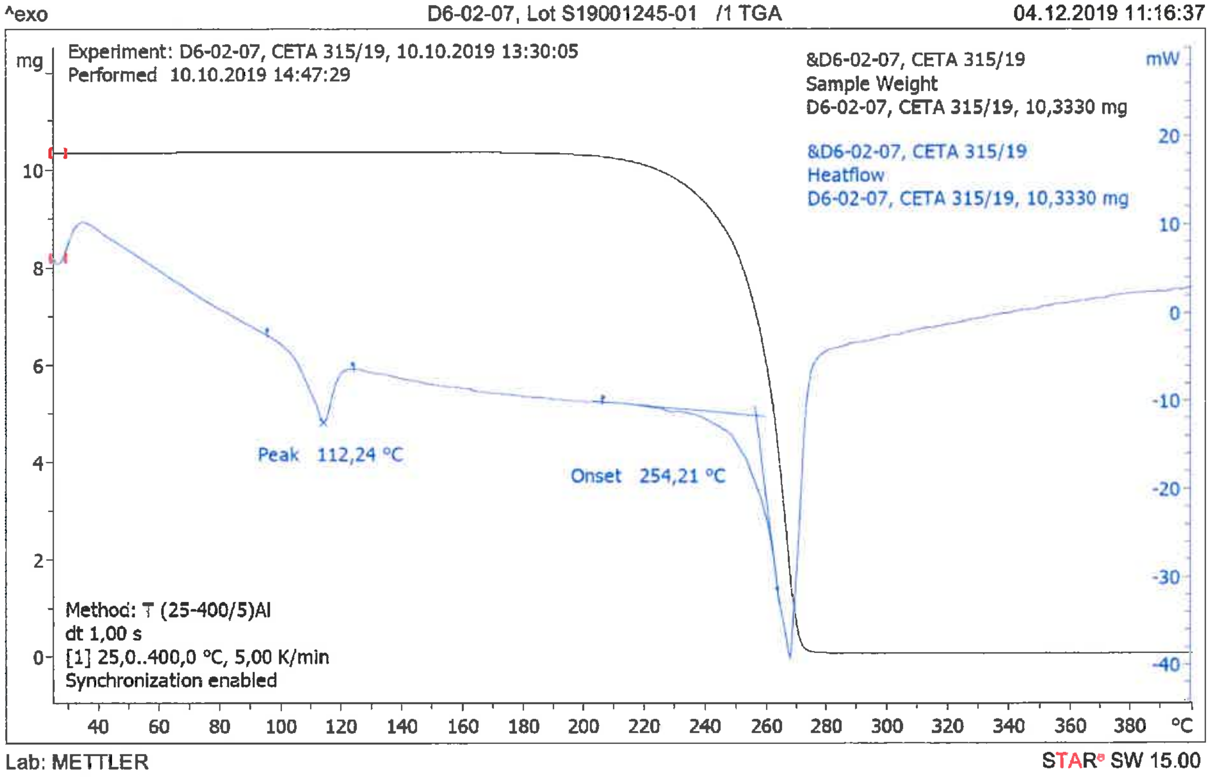

Obrázek č. 5: TGA vzorku D6-02-07, Lot S19001245-01, CETA č. 315/19 (8 týdnů degradovaný) (2. měření)

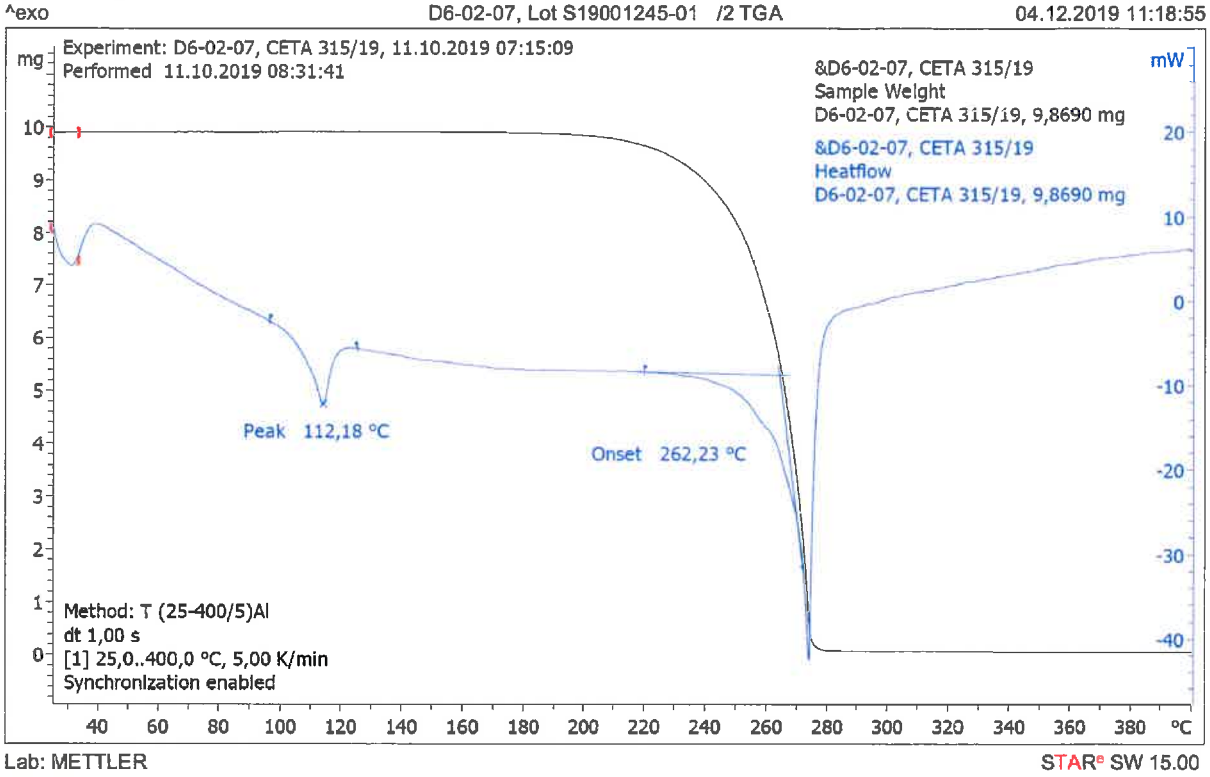

č. 20-0204-61

Název:	**Termická analýza biodegradabilního polymeru**		
Přijetí vzorku:	27. 11. 2019		
Vypracoval:	Z. Křenková	Zkontroloval:	T. Čermák
Zadavatel:	ELLA-CS s.r.o., Milady Horákové 504/45, 500 06 Hradec Králové		

Výsledky:

Byla provedena termická analýza biodegradabilního polymeru:
D6-02-10, Lot S19001245-01, CETA č. 368/19 (16 týdnů degradovaný)

Výsledky diferenciální skenovací kalorimetrie (DSC) jsou uvedeny v tabulce č. 1 (teploty tání a krystalizace, entalpie tání a studené krystalizace, skelný přechod) a v tabulce č. 2 (krystalinita).
Měření DSC jsou zobrazena na obrázcích č. 1 – 3.
Měření termogravimetrické analýzy (TGA) jsou zobrazena na obrázcích č. 4 a 5.

Poznámky:

Analýza byla provedena v souladu s normami:

- ČSN EN ISO 11357-1 Plasty – Diferenciální snímací kalorimetrie (DSC) – Část 1: Základní principy
- ČSN EN ISO 11357-2 Plasty - Diferenciální snímací kalorimetrie (DSC) – Část 2: Stanovení teploty a výšky skoku skelného přechodu
- ČSN EN ISO 11357-3 Plasty - Diferenciální snímací kalorimetrie (DSC) – Část 3: Stanovení teploty a entalpie tání a krystalizace
- ČSN EN ISO 11358-1 Plasty – Termogravimetrie (TG) polymerů – Část 1: Obecné principy

Ing. Michal Bartoš
Vedoucí úseku ANALYTIKA

Signature valid
Digitálně podepsáno
Jméno: Ing. Michal Bartoš
Datum: 04.03.2020 09:59:07

Výzkumný ústav organických syntéz a.s.
č.p. 296,
533 54 Rybitví
CZECH REPUBLIC
Tel: +420 466 822 501
e-mail:analytika@vuos.com
homepage:www.vuos.com
Registrováno: Krajský soud Hradec Králové
oddíl B, vložka 1033
DIČ: CZ60108975

Analyzovaný vzorek:

Název: D6-02-10, Lot S19001245-01, CETA č. 368/19 (16 týdnů degradovaný)
Materiál: Polydioxanon (PDO).
Skladování: Exsikátor, laboratorní teplota

Diferenční skenovací kalorimetrie

Přístrojové vybavení:

DSC823^e Mettler Toledo

- Měřící teplotní rozsah: 203 – 973 K (-70 to 700 °C)
- Rozlišení: přibližně 0.07 µW
- Měřící kelímek: hliníkový kelímek (100µl), zalisovaný víčkem s otvorem vytvořeným speciálním bodlem
- Referenční kelímek: prázdný hliníkový kelímek (100µl), zalisovaný víčkem s otvorem vytvořeným speciálním bodlem

S ohledem na požadavky hodnocení krystalinity byly vzorky navažovány na mikrovahách (součást zařízení TGA/DSC1 Mettler Toledo).

Metoda:

Krok		Teplotní rozsah [°C]	Čas isotermy [min]/ teplotní gradient [°C/min]
1.	isotermický krok	25	5 min
2.	dynamický krok – 1.ohřev	25 – 150	5 °C/min
3.	isotermický krok	150	5 min
4.	dynamický krok – chlazení	150 - -30	5 °C/min
5.	isotermický krok	-30	5 min
6.	dynamický krok – 2.ohřev	-30 - 150	5 °C/min

Termogravimetrická analýza

Přístrojové vybavení:

TGA/DSC1 Mettler Toledo

- Teplotní rozsah: 298 – 1373 K (25 – 1100 °C)
- Citlivost: 0.5 mW
- Teplotní rozlišení: 0.005 K
- Měřící kelímek: hliníkový kelímek (100µl), zalisovaný víčkem s otvorem vytvořeným speciálním bodlem

Metoda:

Krok		Teplotní rozsah [°C]	Teplotní gradient [°C/min]
1.	dynamický krok	25 – 400	5

Průtok inertního plynu (dusík) byl nastaven na cca 80 ml/min.

Hodnocení a závěr:

Diferenční skenovací kalorimetrie

Tabulka č. 1: Termická charakterizace vzorku D6-02-10, Lot S19001245-01, CETA č. 368/19 (16 týdnů degradovaný)

Stanovení č.	Teplota tání			Entalpie tání	Teplota krystalizace		Skelný přechod	Entalpie studené krystalizace	Entalpie studené krystalizace (premelting)	Entalpie tání
	T_m (onset) [°C]	Peak 1 [°C]	Peak 2 [°C]	ΔH_f [J/g]	T_c (onset) [°C]	Peak [°C]	Tg [°C]	ΔH_c [J/g]	ΔH_c [J/g]	ΔH_f [J/g]
	**1.ohřev**			**1.ohřev**	**chlazení**		**2.ohřev**	**2. ohřev**	**2. ohřev**	**2.ohřev**
**1**	99,27	-	110,80	96,12	62,52	50,64	-18,98	8,02	9,91	71,54
**2**	100,21	-	111,57	97,00	62,52	48,82	-19,12	7,95	9,53	75,68
**3**	99,09	-	111,10	101,97	62,89	49,91	-18,04	4,48	10,03	76,44
**Průměr**	**99,5**	**-**	**111,2**	**98,4**	**62,6**	**49,8**	**-18,7**	**6,8**	**9,8**	**74,6**
SD	0,6	-	0,4	3,2	0,2	0,9	0,6	2,0	0,3	2,6

Onset teplota ...... extrapolovaná počáteční teplota (průsečík extrapolované základní čáry tepelného toku s tečnou v inflexním bodu píku)

Výpočty:

$$Xc = \frac{\Delta H_f - \Delta H_c}{\Delta H_{f\,100\%}} \times 100$$

X_c..............................Krystalinita; procentuální podíl krystalické fáze polymeru [%]
ΔH_f............................Entalpie tání; teplo pohlcené v průběhu tání vzorku [J/g]
ΔH_c ....Entalpie studené krystalizace; teplo uvolněné v průběhu studené krystalizace vzorku [J/g]
$\Delta H_{f\,100\%}$..................Teoretická hodnota entalpie tání 100% krystalického polymeru PDO [J/g]; 141,18 J/g (zdroj: Thermal properties and non-isothermal crystallization behavior of biodegradable poly(p-dioxanone)/poly(vinyl alcohol) blends; DOI: 10.1002/pi.1904)

Krystalinita polymeru (Xc) vyhodnocená během 1. ohřevu charakterizuje polymer ("as received") s jeho teplotní a mechanickou historií (vliv výrobního postupu, skladování atd.). Krystalinita vyhodnocená během 2. ohřevu charakterizuje polymer po vymazání jeho teplotní i mechanické historie a slouží především ke srovnávání polymerních materiálů vzájemně.

Tabulka č. 2: Krystalinita vzorku D6-02-10, Lot S19001245-01, CETA č. 368/19 (16 týdnů degradovaný) vyhodnocená během 1. a 2. ohřevu

Stanovení č.	Krystalinita, X_c [%]	
	1.ohřev	2. ohřev
**1**	68,08	37,97
**2**	68,71	41,22
**3**	72,23	43,87
**Průměr**	**69,7**	**41,0**
SD	2,2	3,0

Obrázek č. 1: DSC vzorku D6-02-10, Lot S19001245-01, CETA č. 368/19 (16 týdnů degradovaný) (1. měření)

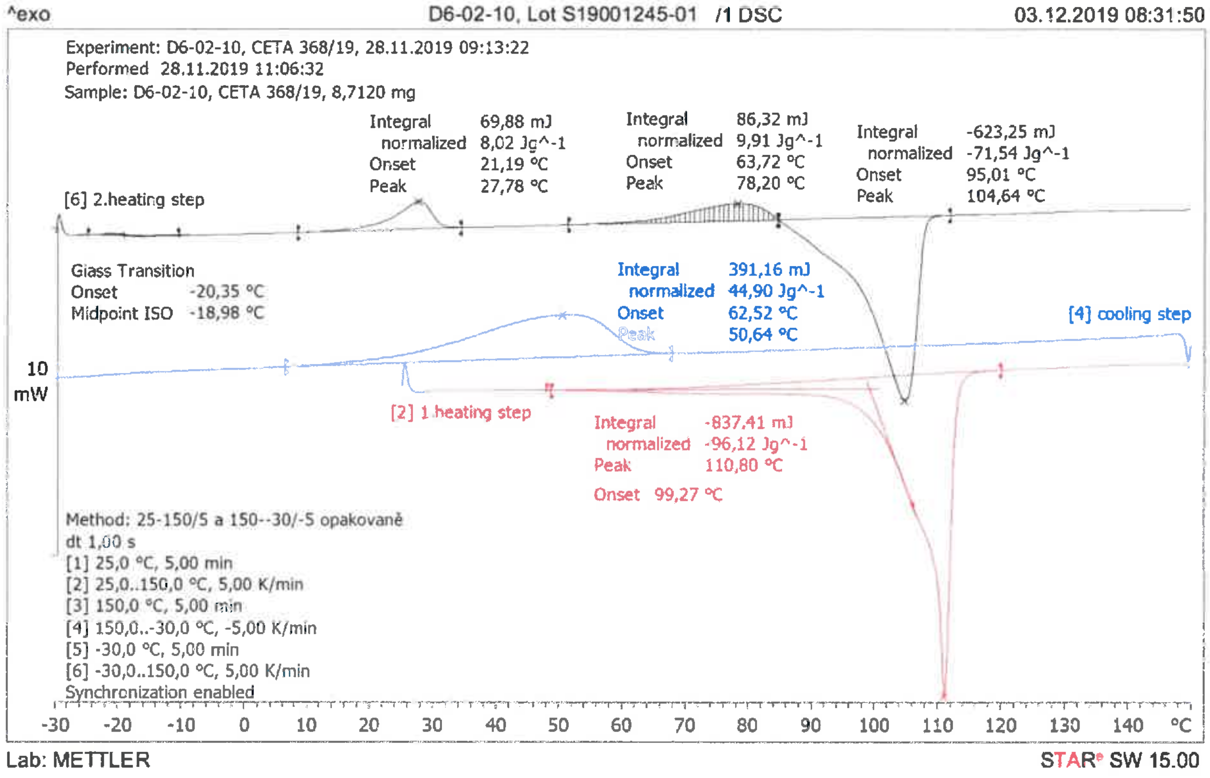

Obrázek č. 2: DSC vzorku D6-02-10, Lot S19001245-01, CETA č. 368/19 (16 týdnů degradovaný) (2. měření)

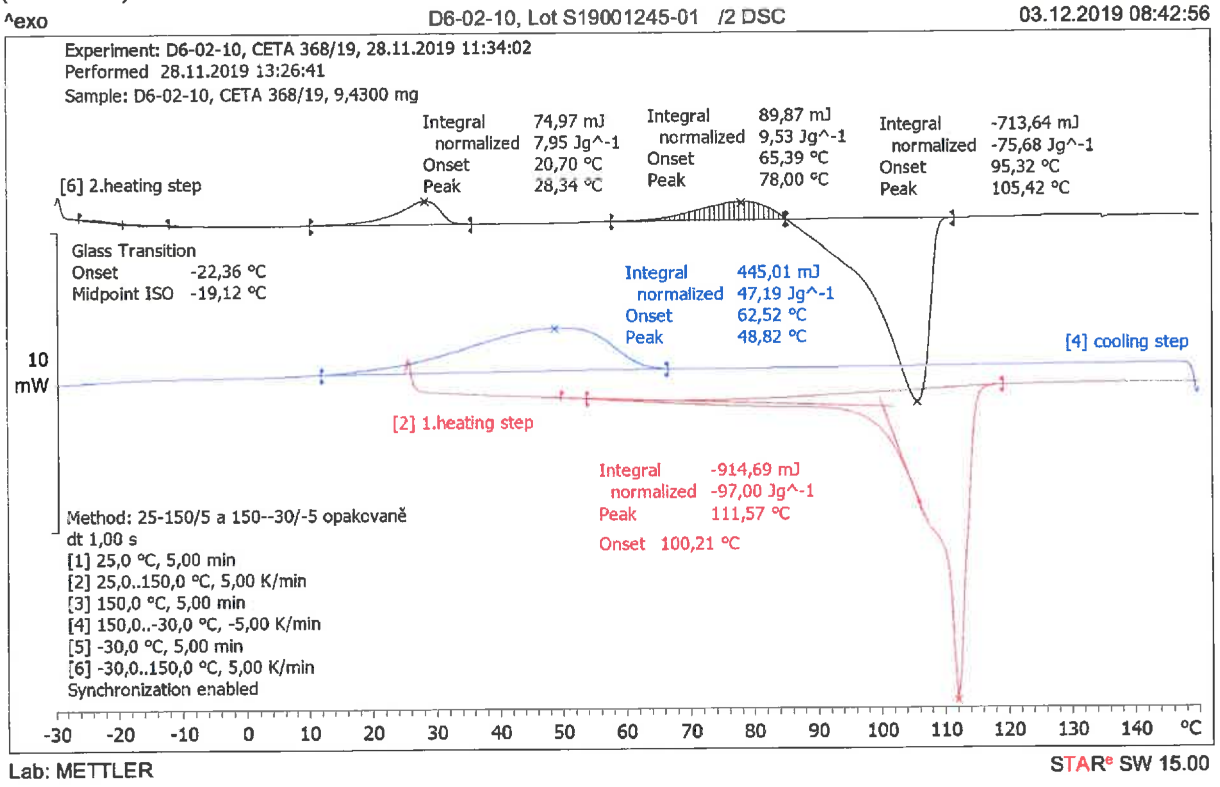

Obrázek č. 3: DSC vzorku D6-02-10, Lot S19001245-01, CETA č. 368/19 (16 týdnů degradovaný) (3. měření)

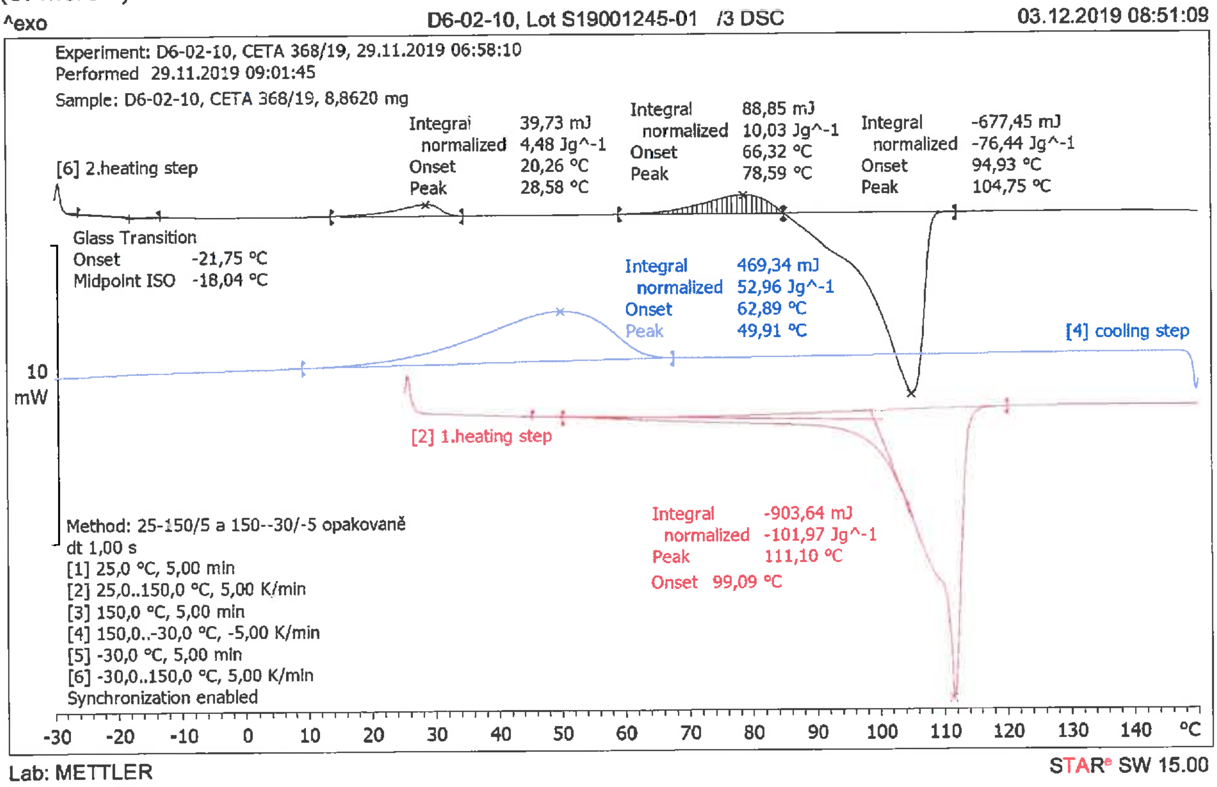

Termogravimetrická analýza

Na obrázcích č. 4 a 5 jsou zobrazena měření TGA u vzorku D6-02-10, Lot S19001245-01, CETA č. 368/19 (16 týdnů degradovaný). Na hmotnostní křivce (černá křivka) nebyl zaznamenán žádný hmotnostní úbytek od 25 °C do 150 °C. Od cca 150 °C začíná termický rozklad polymeru. Na křivce tepelného toku (modrá křivka) byl zaznamenán endotermický pík charakterizovaný teplotou píku 110,99 (1. měření) a 111,07 °C (2. měření), odpovídající tání polymeru. Druhý endotermický pík odpovídá termickému rozkladu polymeru a je charakterizovaný onset teplotou 212,11 °C (1. měření) a 213,43 °C (2. měření).

Obrázek č. 4: TGA vzorku D6-02-10, Lot S19001245-01, CETA č. 368/19 (16 týdnů degradovaný) (1. měření)

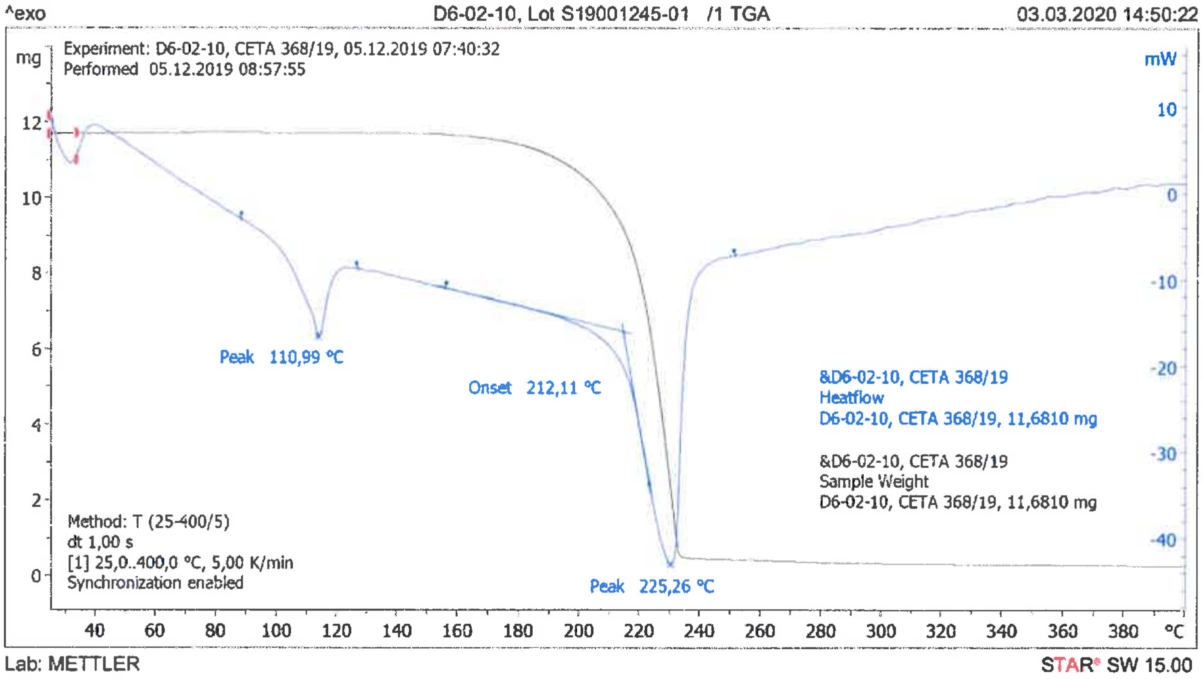

Obrázek č. 5: TGA vzorku D6-02-10, Lot S19001245-01, CETA č. 368/19 (16 týdnů degradovaný) (2. měření)

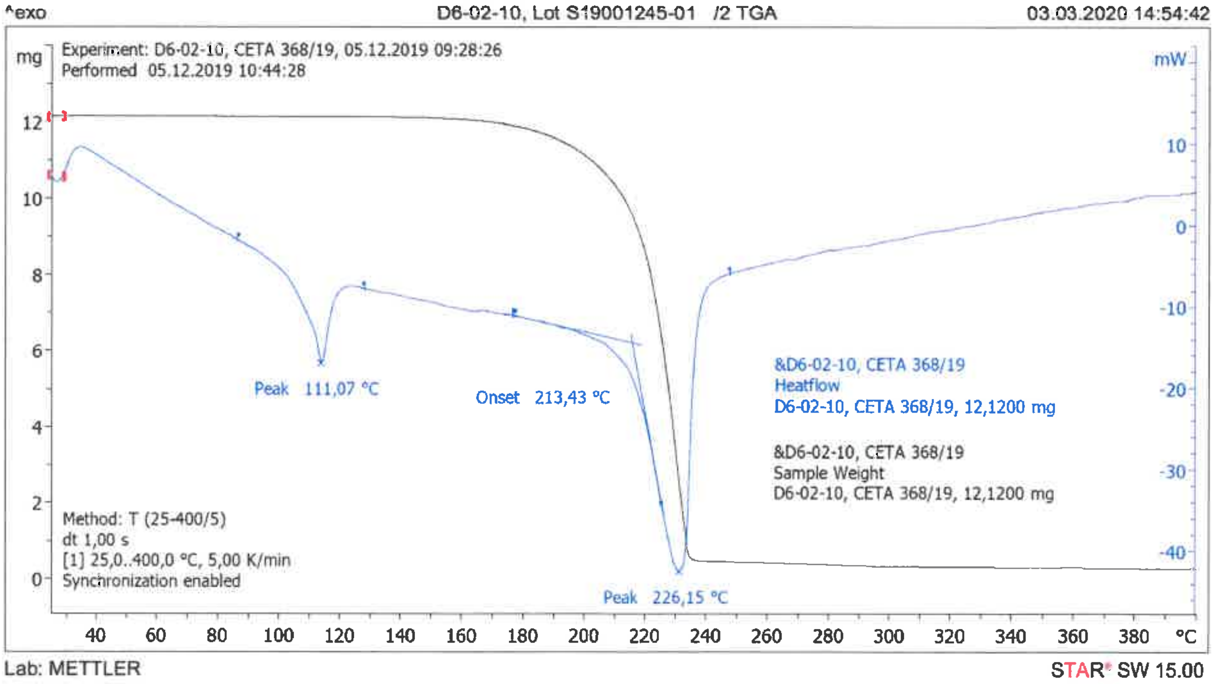

č. 20-0324-61

Název:	**Termická analýza biodegradabilního polymeru**		
Přijetí vzorku:	24. 1. 2020		
Vypracoval:	T. Čermák	Zkontroloval:	J. Vaněček
Zadavatel:	ELLA-CS s.r.o., Milady Horákové 504/45, 500 06 Hradec Králové		

Výsledky:

Byla provedena termická analýza biodegradabilního polymeru:
D6-02-13, Lot S19001245-01, CETA č. 21/20 (24 týdnů degradovaný)

Výsledky diferenciální skenovací kalorimetrie (DSC) jsou uvedeny v tabulce č. 1 (teploty tání a krystalizace, entalpie tání a studené krystalizace) a v tabulce č. 2 (krystalinita).
Měření DSC jsou zobrazena na obrázcích č. 1 – 3.
Měření termogravimetrické analýzy (TGA) jsou zobrazena na obrázcích č. 4 a 5.

Poznámky:

Analýza byla provedena v souladu s normami:
- ČSN EN ISO 11357-1 Plasty – Diferenciální snímací kalorimetrie (DSC) – Část 1: Základní principy
- ČSN EN ISO 11357-2 Plasty - Diferenciální snímací kalorimetrie (DSC) – Část 2: Stanovení teploty a výšky skoku skelného přechodu
- ČSN EN ISO 11357-3 Plasty - Diferenciální snímací kalorimetrie (DSC) – Část 3: Stanovení teploty a entalpie tání a krystalizace
- ČSN EN ISO 11358-1 Plasty – Termogravimetrie (TG) polymerů – Část 1: Obecné principy

Ing. Michal Bartoš
Vedoucí úseku ANALYTIKA

Digitálně podepsáno
Jméno: Ing. Michal Bartoš
Datum: 14.04.2020 09:38:15

Analyzovaný vzorek:

Název: D6-02-13, Lot S19001245-01, CETA č. 21/20 (24 týdnů degradovaný)
Materiál: Polydioxanon (PDO).
Skladování: Exsikátor, laboratorní teplota

Diferenční skenovací kalorimetrie

Přístrojové vybavení:

DSC823^{e} Mettler Toledo

- Měřící teplotní rozsah: 203 – 973 K (-70 to 700 °C)
- Rozlišení: přibližně 0.07 μW
- Měřící kelímek: hliníkový kelímek (100μl), zalisovaný víčkem s otvorem vytvořeným speciálním bodlem
- Referenční kelímek: prázdný hliníkový kelímek (100μl), zalisovaný víčkem s otvorem vytvořeným speciálním bodlem

S ohledem na požadavky hodnocení krystalinity byly vzorky navažovány na mikrovahách (součást zařízení TGA/DSC1 Mettler Toledo).

Metoda:

Krok		Teplotní rozsah [°C]	Čas isotermy [min]/ teplotní gradient [°C/min]
1.	isotermický krok	25	5 min
2.	dynamický krok – 1.ohřev	25 – 150	5 °C/min
3.	isotermický krok	150	5 min
4.	dynamický krok – chlazení	150 - -40	5 °C/min
5.	isotermický krok	-40	5 min
6.	dynamický krok – 2.ohřev	-40 - 150	5 °C/min

Termogravimetrická analýza

Přístrojové vybavení:

TGA/DSC1 Mettler Toledo

- Teplotní rozsah: 298 – 1373 K (25 – 1100 °C)
- Citlivost: 0.5 mW
- Teplotní rozlišení: 0.005 K
- Měřící kelímek: hliníkový kelímek (100μl), zalisovaný víčkem s otvorem vytvořeným speciálním bodlem

Metoda:

Krok		Teplotní rozsah [°C]	Teplotní gradient [°C/min]
1.	dynamický krok	25 – 400	5

Průtok inertního plynu (dusík) byl nastaven na cca 80 ml/min.

Hodnocení a závěr:

Diferenční skenovací kalorimetrie

Tabulka č. 1: Termická charakterizace vzorku D6-02-13, Lot S19001245-01, CETA č. 21/20 (24 týdnů degradovaný)

Stanovení č.	Teplota tání			Entalpie tání	Teplota krystalizace		Skelný přechod	Entalpie studené krystalizace	Entalpie studené krystalizace (premelting)	Entalpie tání
	**T_m (onset) [°C]**	**Peak 1 [°C]**	**Peak 2 [°C]**	**ΔH_f [J/g]**	**T_c (onset) [°C]**	**Peak [°C]**	**Tg [°C]**	**ΔH_c [J/g]**	**ΔH_c [J/g]**	**ΔH_f [J/g]**
	**1.ohřev**			**1.ohřev**	**chlazení**		**2.ohřev**	**2. ohřev**	**2. ohřev**	**2.ohřev**
**1**	99,98	-	111,95	77,55	58,35	45,30	-	-	9,15	63,73
**2**	99,87	-	111,11	86,99	58,18	45,57	-	-	9,25	65,91
**3**	98,91	-	110,99	86,16	58,43	45,73	-	-	8,99	67,45
**Průměr**	**99,6**	**-**	**111,4**	**83,6**	**58,3**	**45,5**	**-**	**-**	**9,1**	**65,7**
SD	0,6	-	0,5	5,2	0,1	0,2	-	-	0,1	1,9

Onset teplota ...... extrapolovaná počáteční teplota (průsečík extrapolované základní čáry tepelného toku s tečnou v inflexním bodu píku)

Výpočty:

$$Xc = \frac{\Delta H_f - \Delta H_c}{\Delta H_{f\,100\%}} \times 100$$

X_c..............................Krystalinita; procentuální podíl krystalické fáze polymeru [%]
ΔH_f............................Entalpie tání; teplo pohlcené v průběhu tání vzorku [J/g]
ΔH_c ....Entalpie studené krystalizace; teplo uvolněné v průběhu studené krystalizace vzorku [J/g]
$\Delta H_{f\,100\%}$...................Teoretická hodnota entalpie tání 100% krystalického polymeru PDO [J/g]; 141,18 J/g (zdroj: Thermal properties and non-isothermal crystallization behavior of biodegradable poly(p-dioxanone)/poly(vinyl alcohol) blends; DOI: 10.1002/pi.1904)

Krystalinita polymeru (Xc) vyhodnocená během 1. ohřevu charakterizuje polymer ("as received") s jeho teplotní a mechanickou historií (vliv výrobního postupu, skladování atd.). Krystalinita vyhodnocená během 2. ohřevu charakterizuje polymer po vymazání jeho teplotní i mechanické historie a slouží především ke srovnávání polymerních materiálů vzájemně.

Tabulka č. 2: Krystalinita vzorku D6-02-13, Lot S19001245-01, CETA č. 21/20 (24 týdnů degradovaný) vyhodnocená během 1. a 2. ohřevu

**Stanovení č.**	**Krystalinita, X_c [%]**	
	**1. ohřev**	**2. ohřev**
**1**	54,93	38,66
**2**	61,62	40,13
**3**	61,03	41,41
**Průměr**	**59,2**	**40,1**
SD	3,7	1,4

Obrázek č. 1: DSC vzorku D6-02-13, Lot S19001245-01, CETA č. 21/20 (24 týdnů degradovaný) (1. měření)

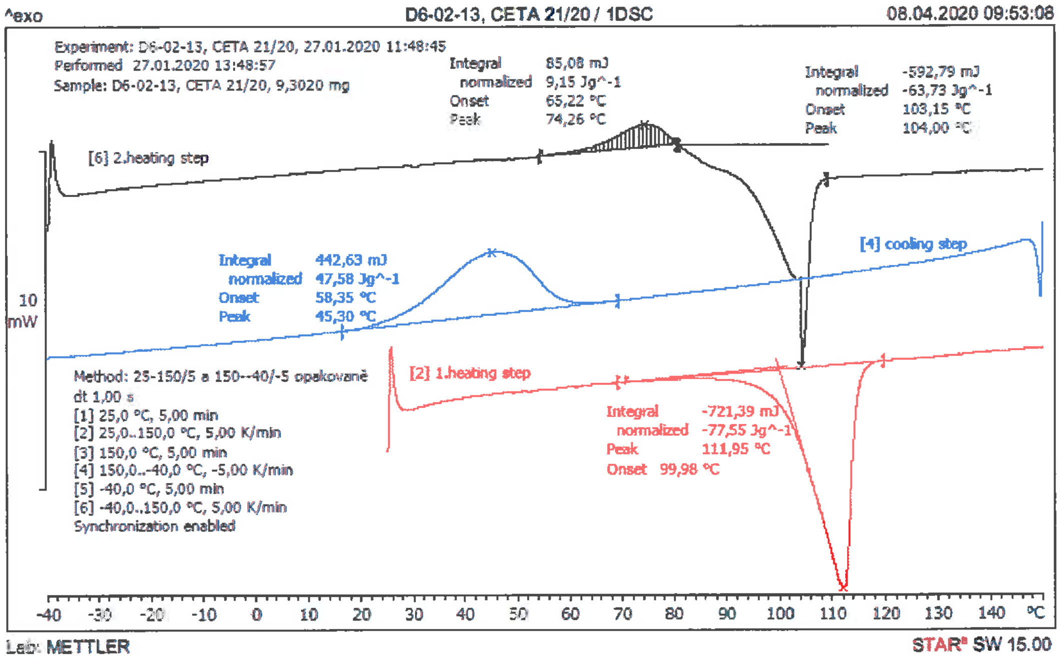

Obrázek č. 2: DSC vzorku D6-02-13, Lot S19001245-01, CETA č. 21/20 (24 týdnů degradovaný) (2. měření)

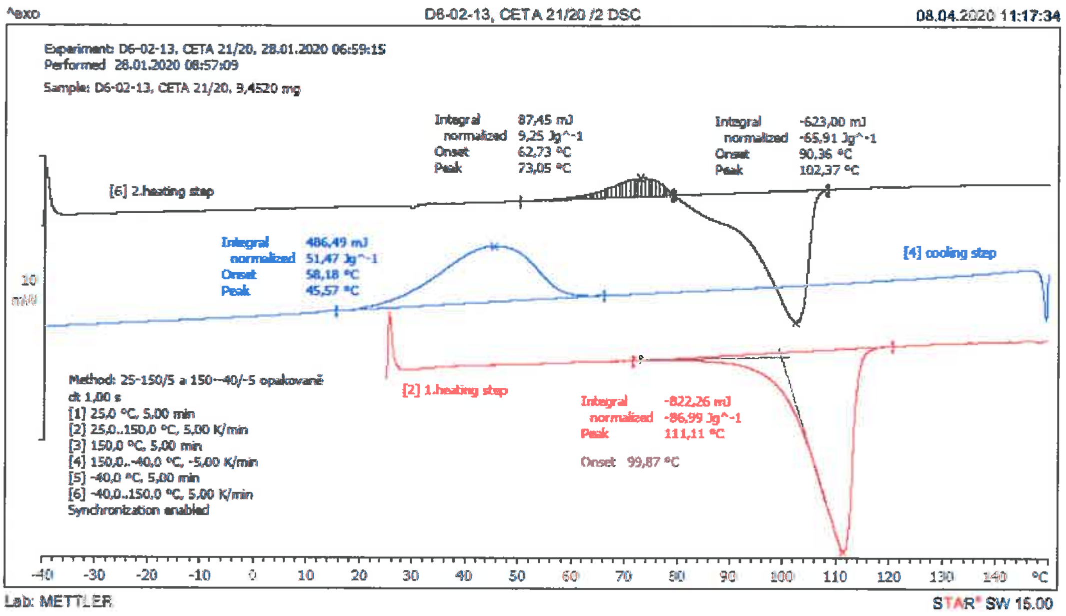

Obrázek č. 3: DSC vzorku D6-02-13, Lot S19001245-01, CETA č. 21/20 (24 týdnů degradovaný) (3. měření)

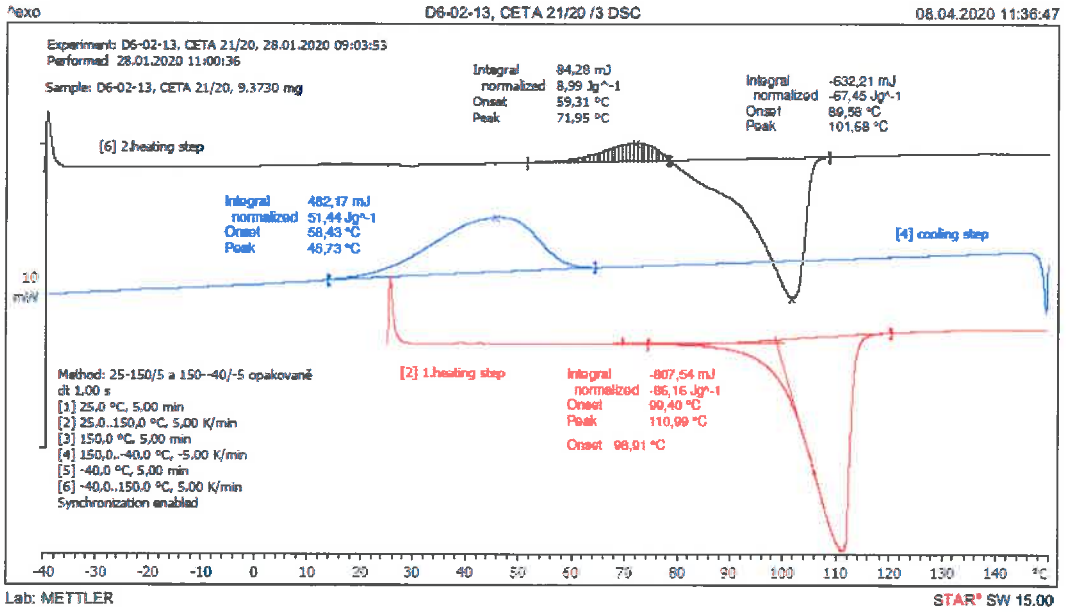

Termogravimetrická analýza

Na obrázcích č. 4 a 5 jsou zobrazena měření TGA u vzorku D6-02-13, Lot S19001245-01, CETA č. 21/20 (24 týdnů degradovaný). Na hmotnostní křivce (černá křivka) nebyl zaznamenán žádný hmotnostní úbytek od 25 °C do 150 °C. Od cca 150 °C začíná termický rozklad polymeru. Na křivce tepelného toku (modrá křivka) byl zaznamenán endotermický pík charakterizovaný teplotou píku 110,13 (1. měření) a 110,64 °C (2. měření), odpovídající tání polymeru. Druhý endotermický pík odpovídá termickému rozkladu polymeru a je charakterizovaný onset teplotou 220,03 °C (1. měření) a 220,34 °C (2. měření).

Obrázek č. 4: TGA vzorku D6-02-13, Lot S19001245-01, CETA č. 21/20 (24 týdnů degradovaný) (1. měření)

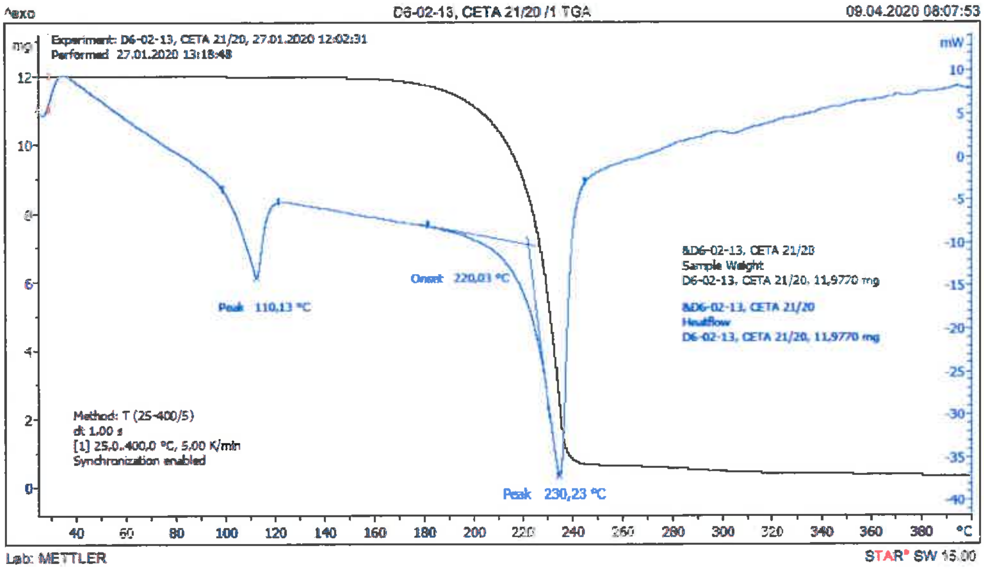

Obrázek č. 5: TGA vzorku D6-02-13, Lot S19001245-01, CETA č. 21/20 (24 týdnů degradovaný) (2. měření)

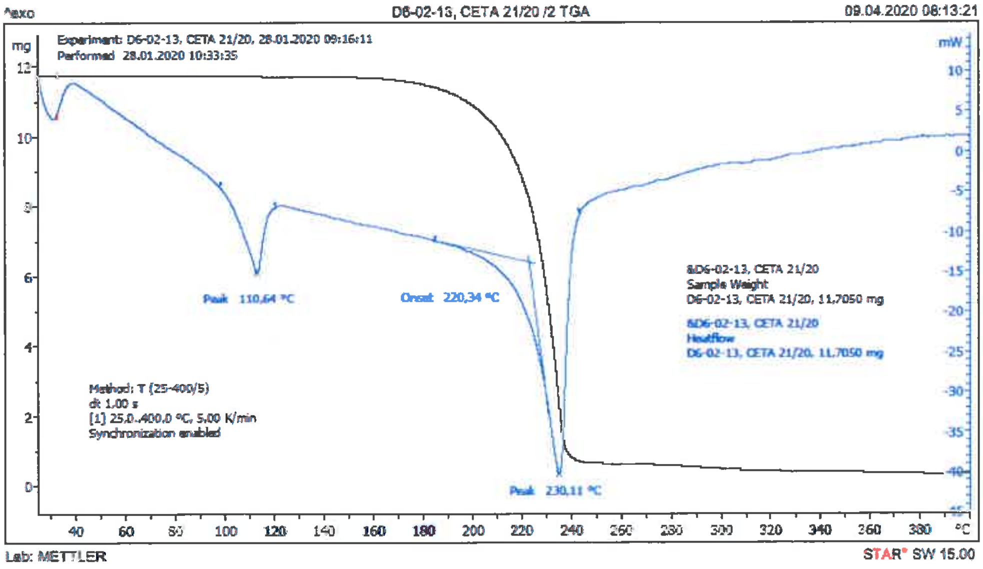

Supplement: Supplementary file 1 [file materials-14-05462-s001.zip › Protocol S1 - DSC measurement protocols.pdf]
