# Supplementary figures and images for: Raman Spectroscopy as a Novel Method for the Characterization of Polydioxanone Medical Stents Biodegradation
Source: Materials (Basel). 2021 Sep 21;14(18):5462. doi: 10.3390/ma14185462 (PMC8467320; doi:10.3390/ma14185462)

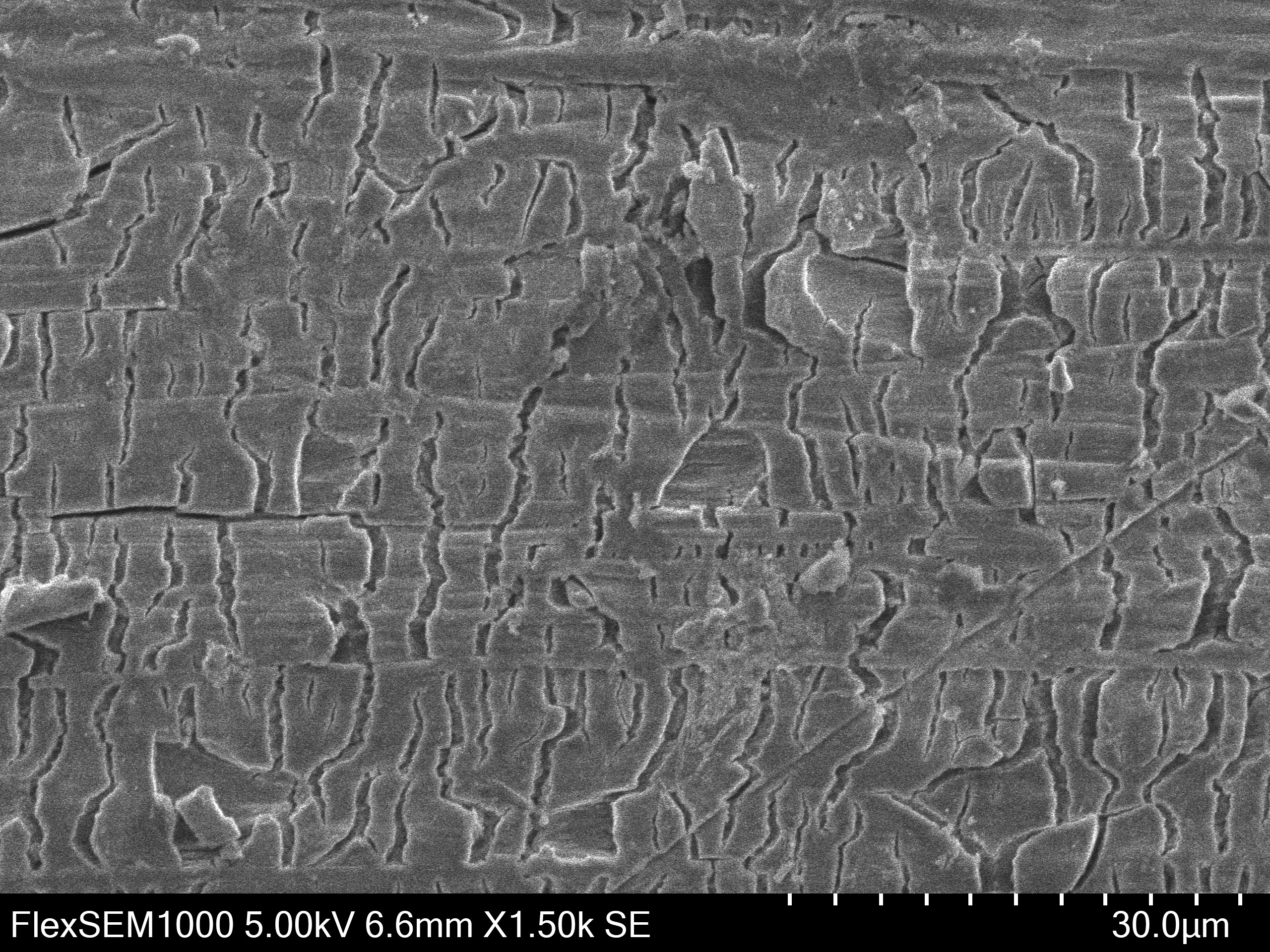

Supplement: Supplementary file 1 [file materials-14-05462-s001.zip › Gallery S1 SEM images of stent surface cracks/16weeks_01_x1500_SE.jpg]

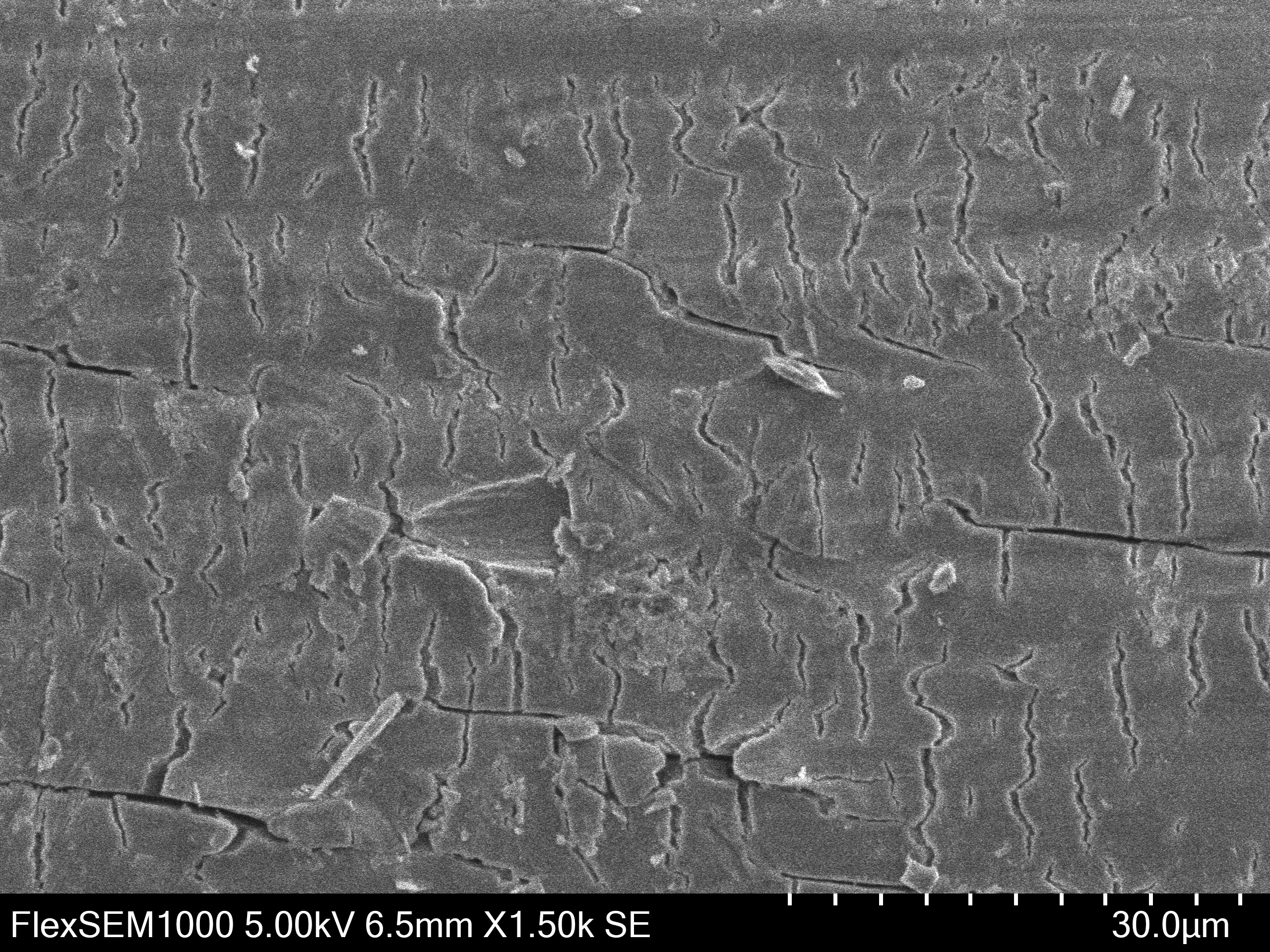

Supplement: Supplementary file 1 [file materials-14-05462-s001.zip › Gallery S1 SEM images of stent surface cracks/16weeks_02_x1500_SE.jpg]

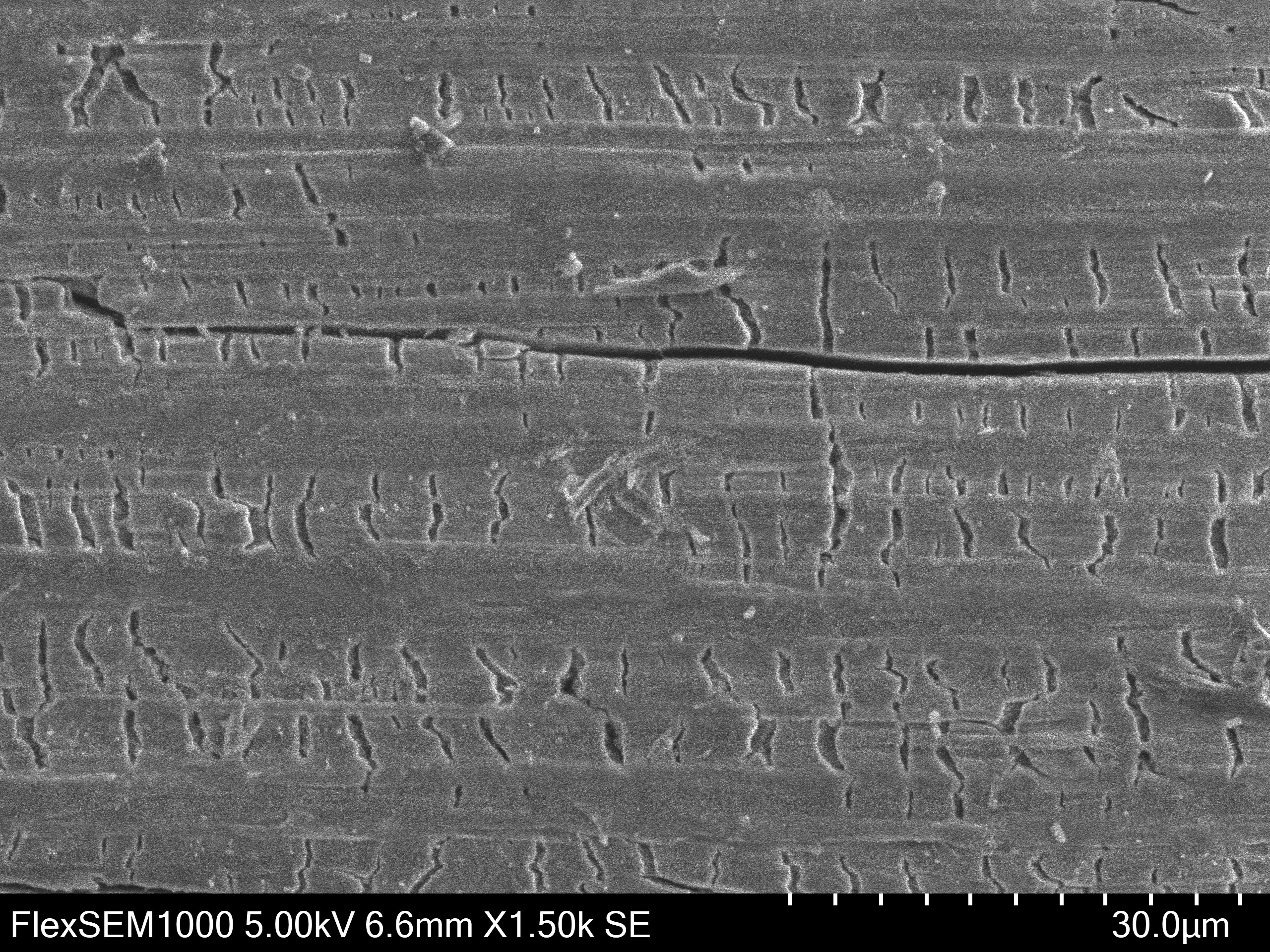

Supplement: Supplementary file 1 [file materials-14-05462-s001.zip › Gallery S1 SEM images of stent surface cracks/16weeks_03_x1500_SE.jpg]

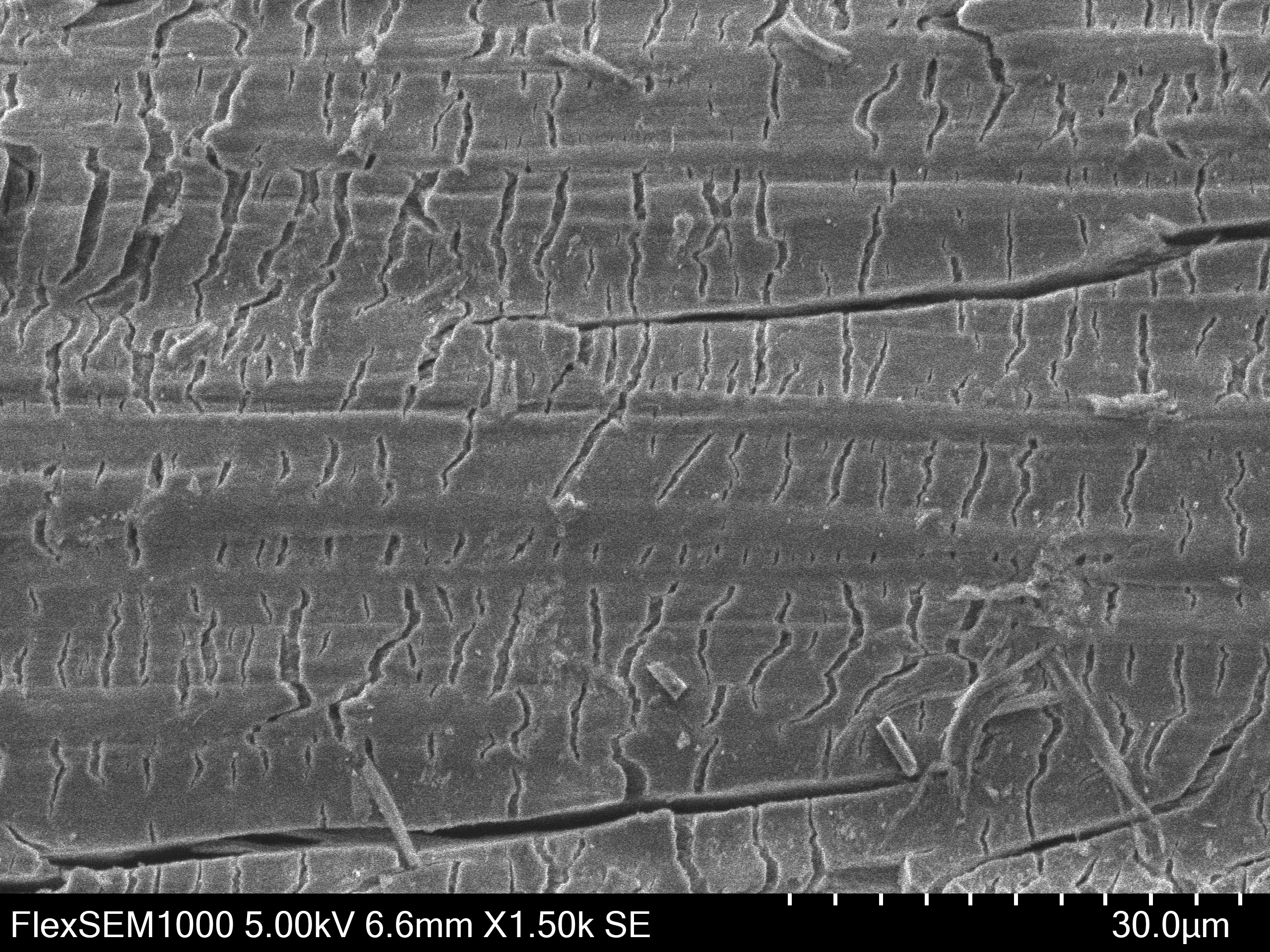

Supplement: Supplementary file 1 [file materials-14-05462-s001.zip › Gallery S1 SEM images of stent surface cracks/16weeks_04_x1500_SE.jpg]

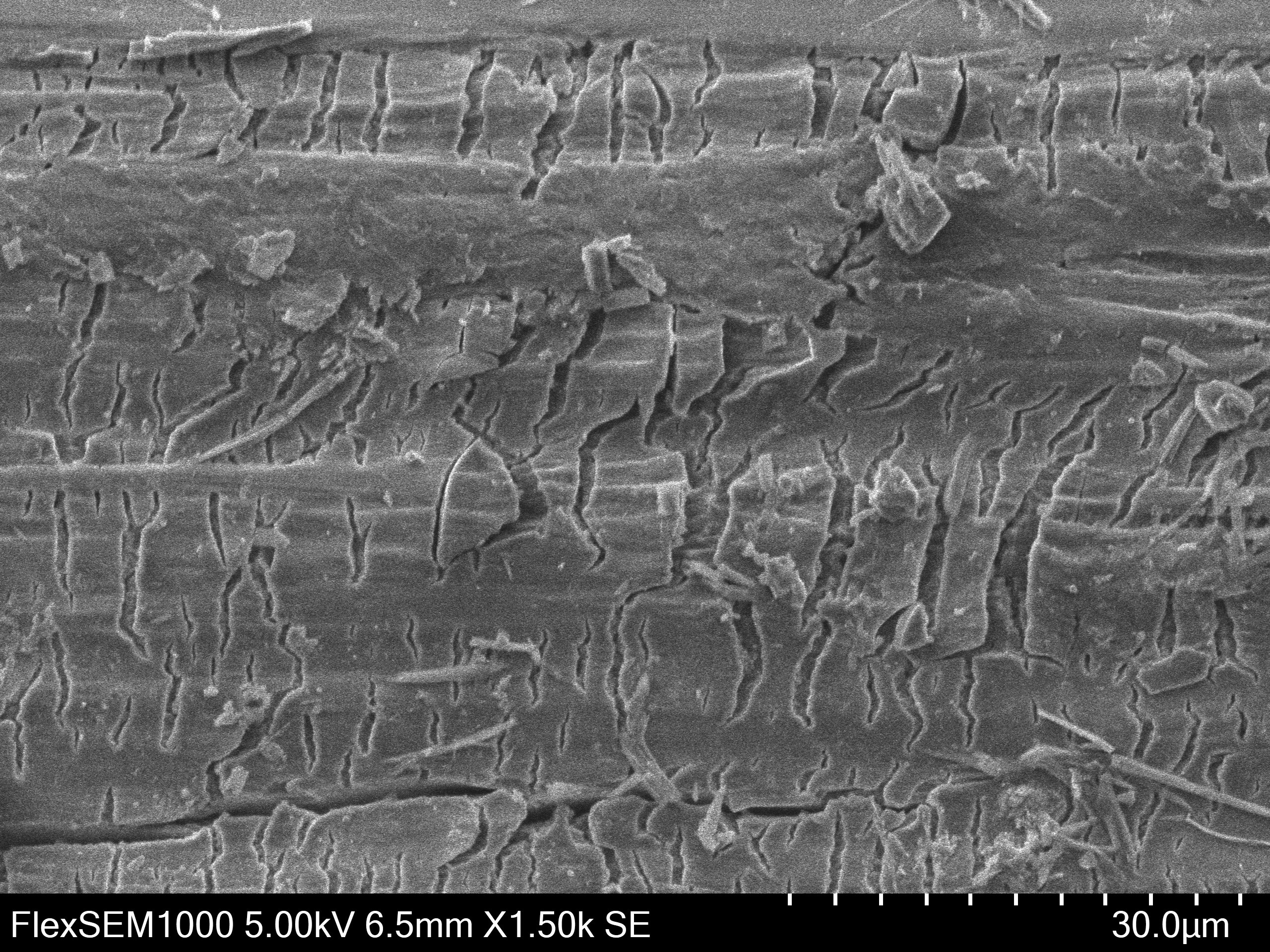

Supplement: Supplementary file 1 [file materials-14-05462-s001.zip › Gallery S1 SEM images of stent surface cracks/16weeks_05_x1500_SE.jpg]

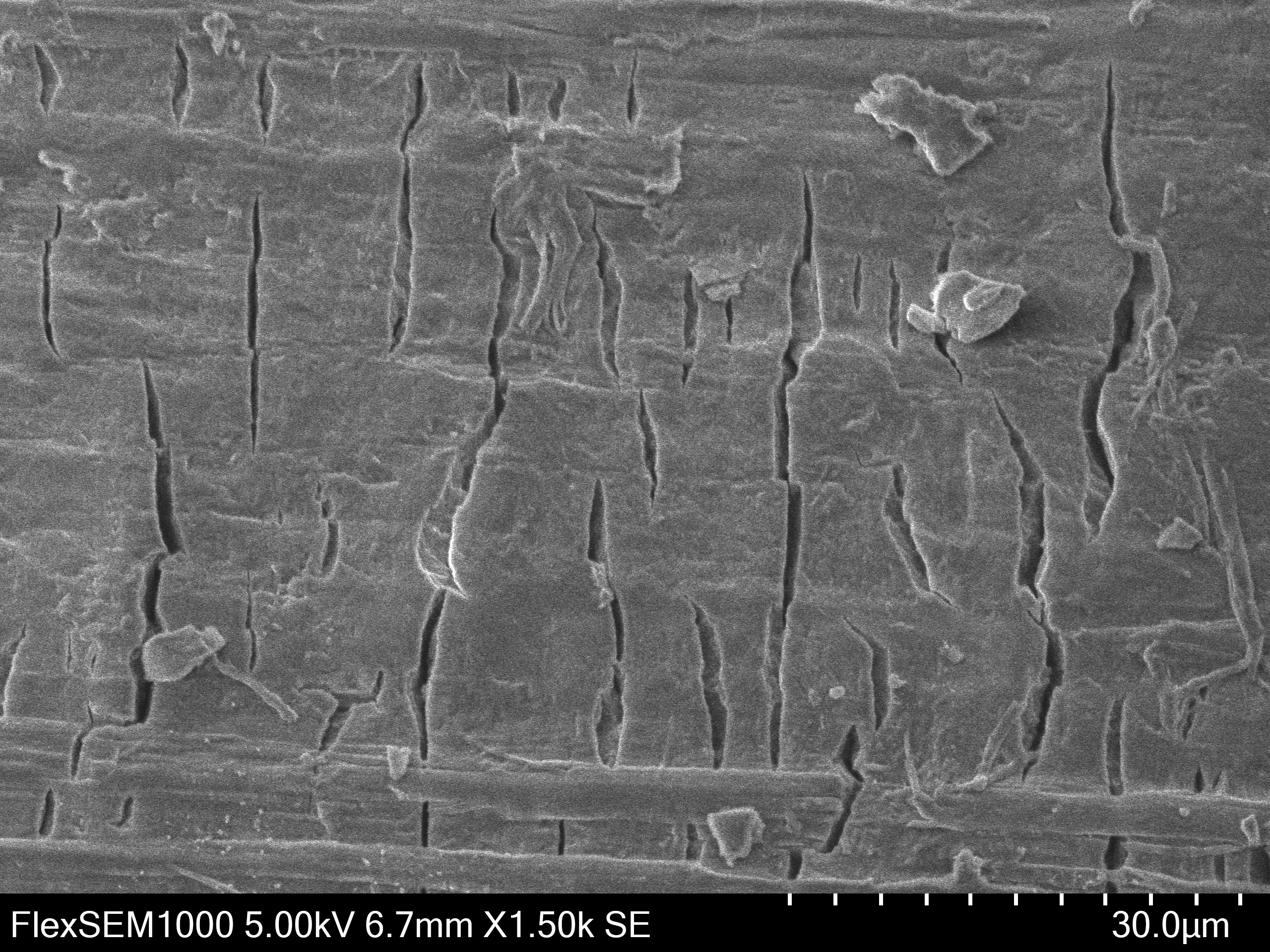

Supplement: Supplementary file 1 [file materials-14-05462-s001.zip › Gallery S1 SEM images of stent surface cracks/24weeks_01_x1500_SE.jpg]

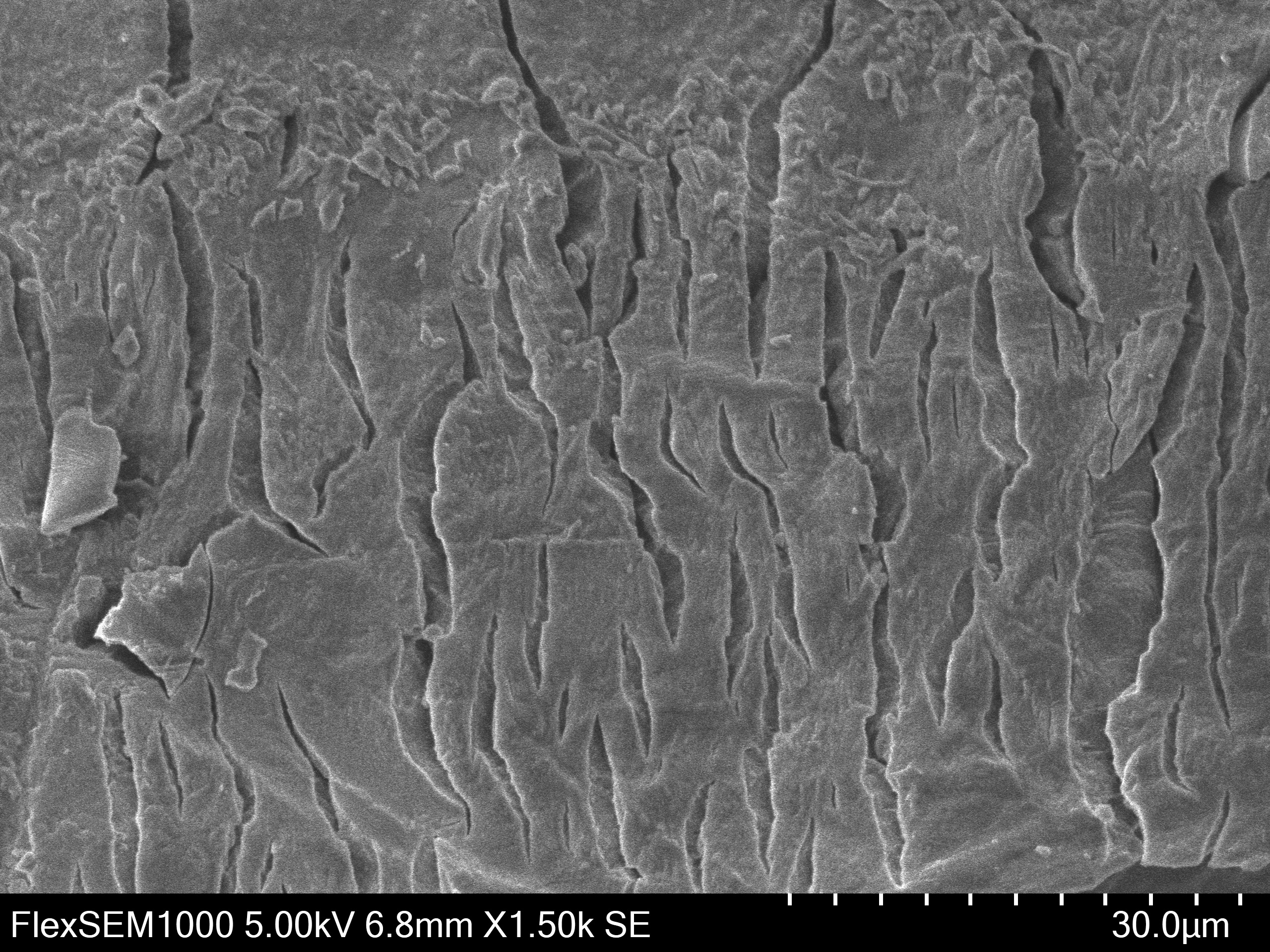

Supplement: Supplementary file 1 [file materials-14-05462-s001.zip › Gallery S1 SEM images of stent surface cracks/24weeks_02_x1500_SE.jpg]

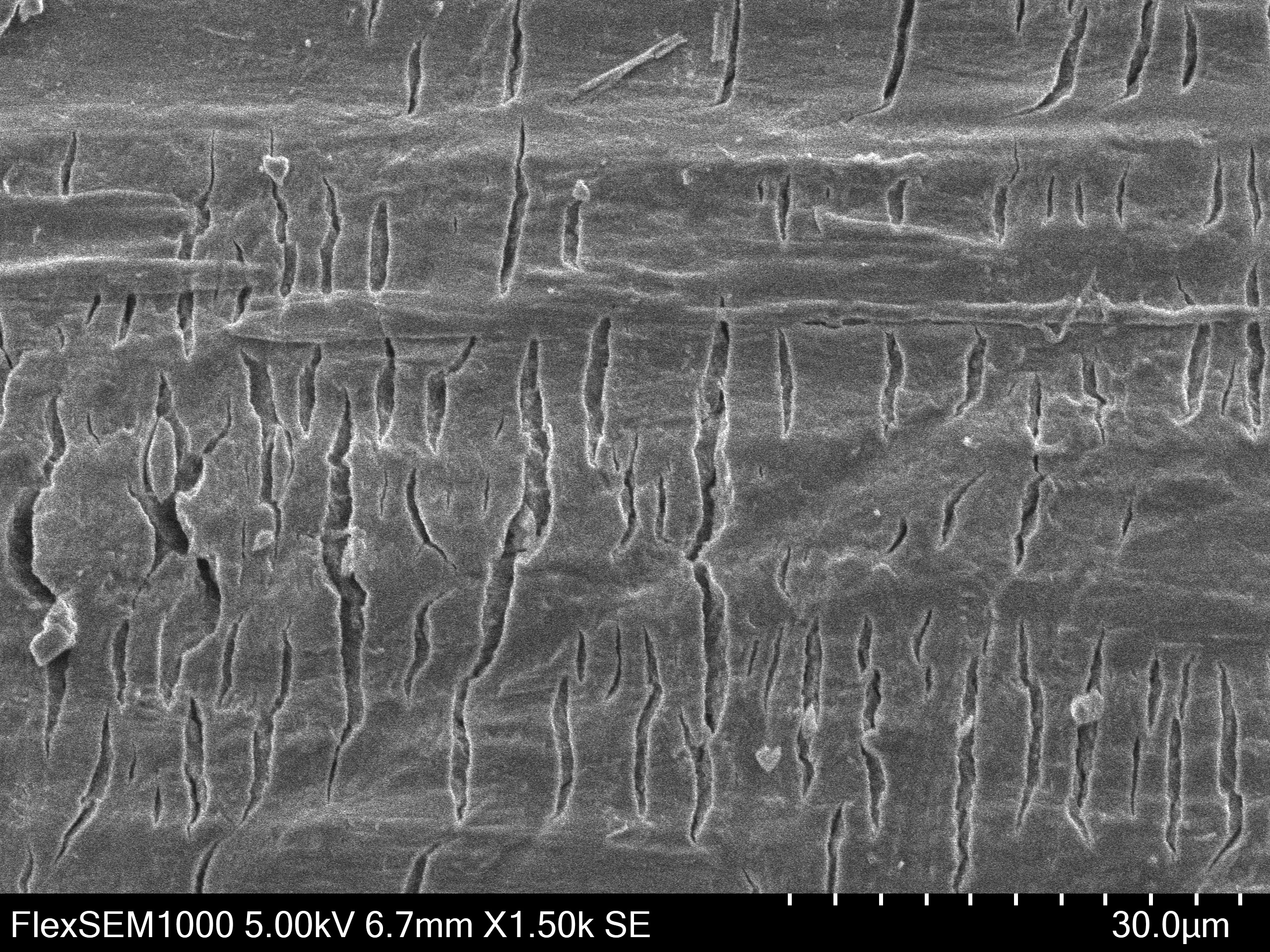

Supplement: Supplementary file 1 [file materials-14-05462-s001.zip › Gallery S1 SEM images of stent surface cracks/24weeks_03_x1500_SE.jpg]

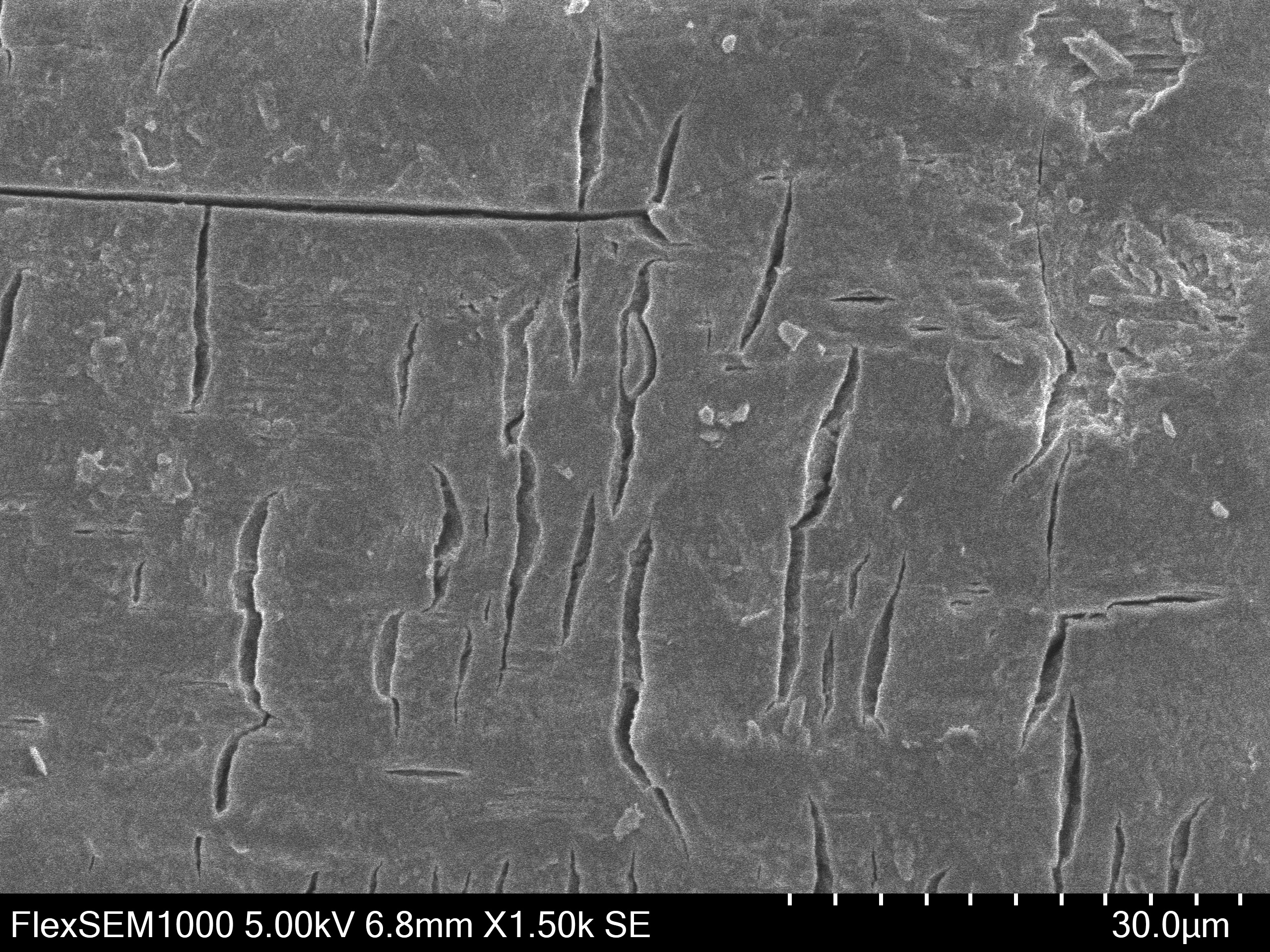

Supplement: Supplementary file 1 [file materials-14-05462-s001.zip › Gallery S1 SEM images of stent surface cracks/24weeks_04_x1500_SE.jpg]

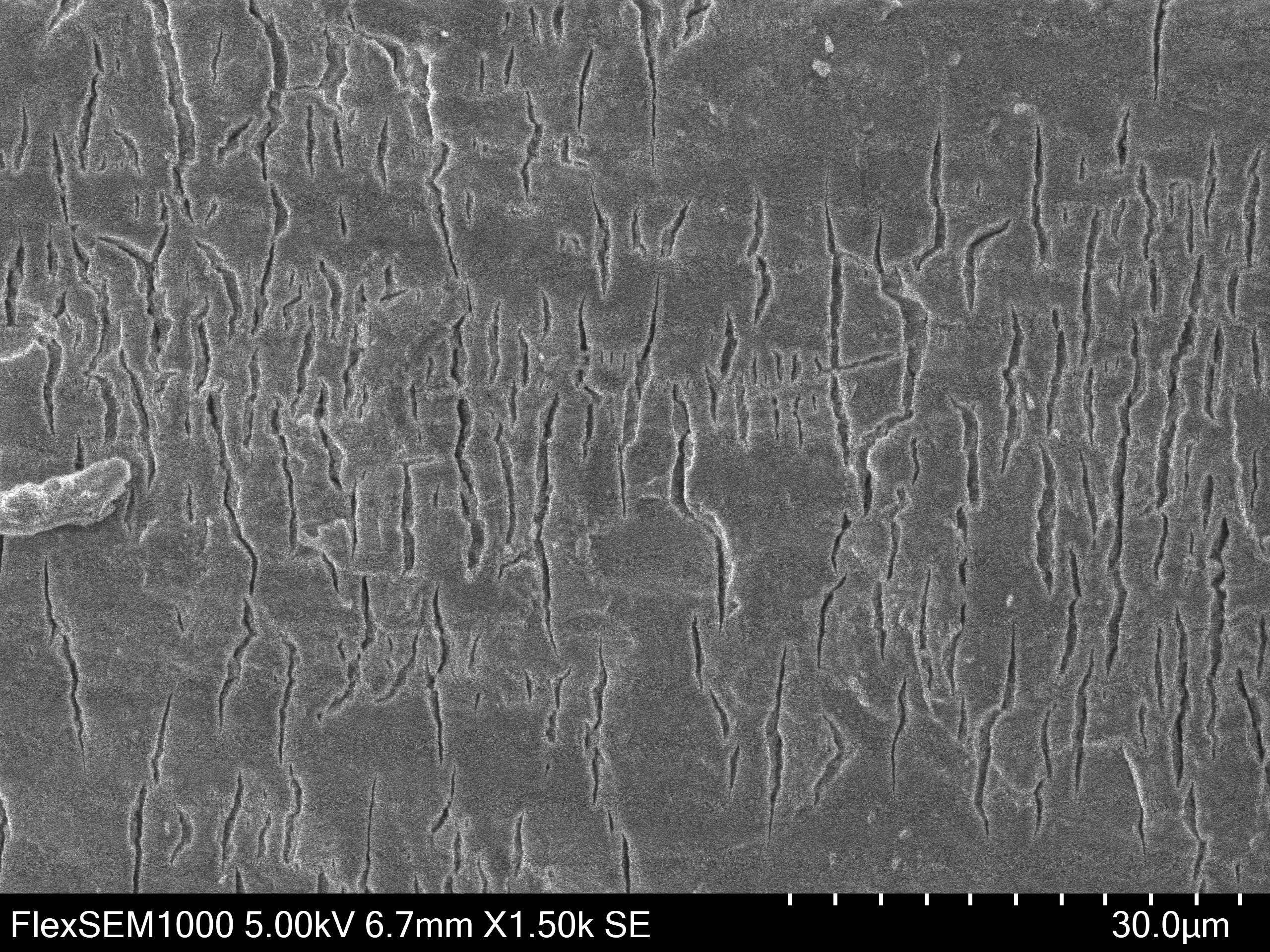

Supplement: Supplementary file 1 [file materials-14-05462-s001.zip › Gallery S1 SEM images of stent surface cracks/24weeks_05_x1500_SE.jpg]

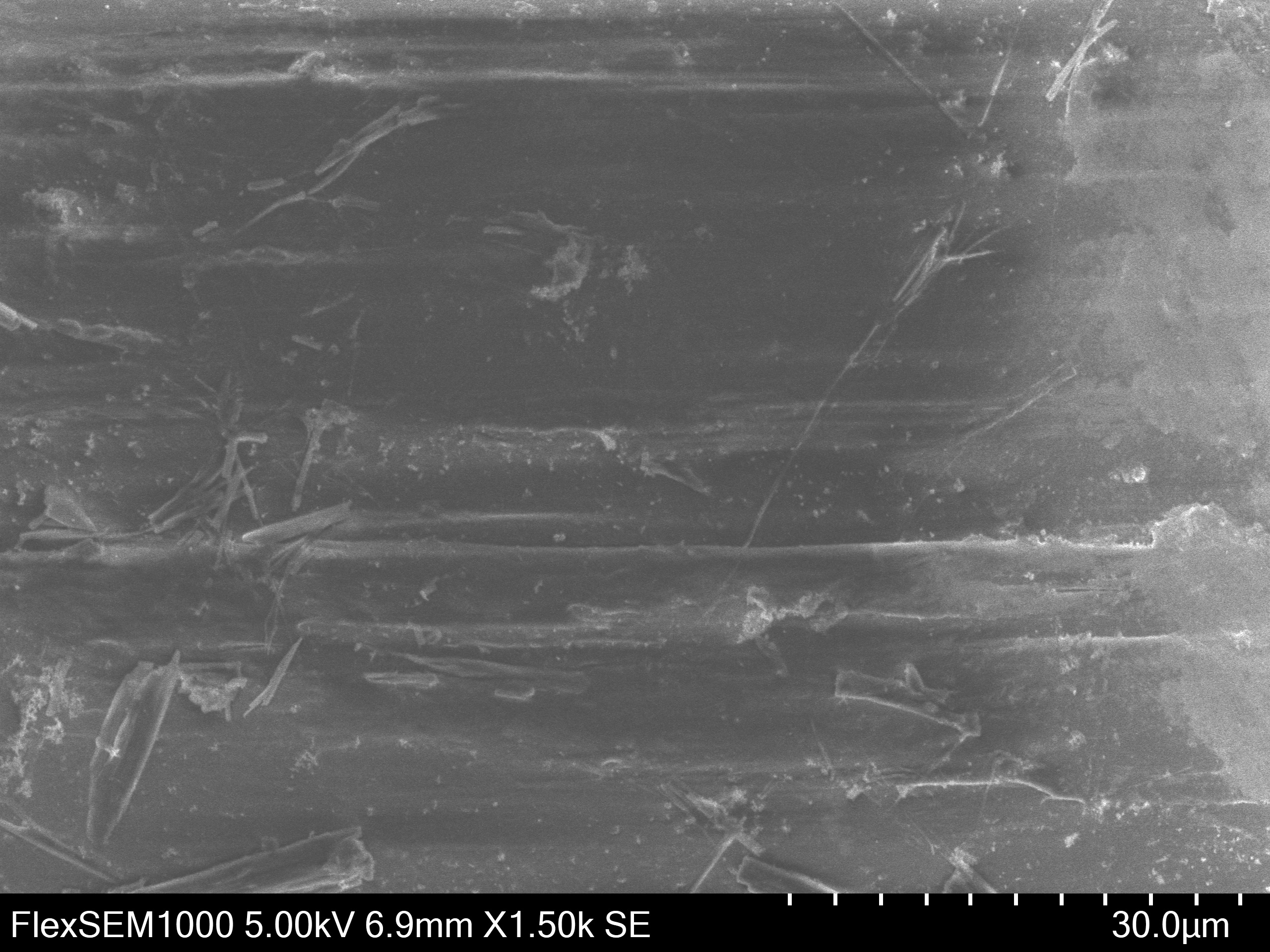

Supplement: Supplementary file 1 [file materials-14-05462-s001.zip › Gallery S1 SEM images of stent surface cracks/4weeks_01_x1500_SE.jpg]

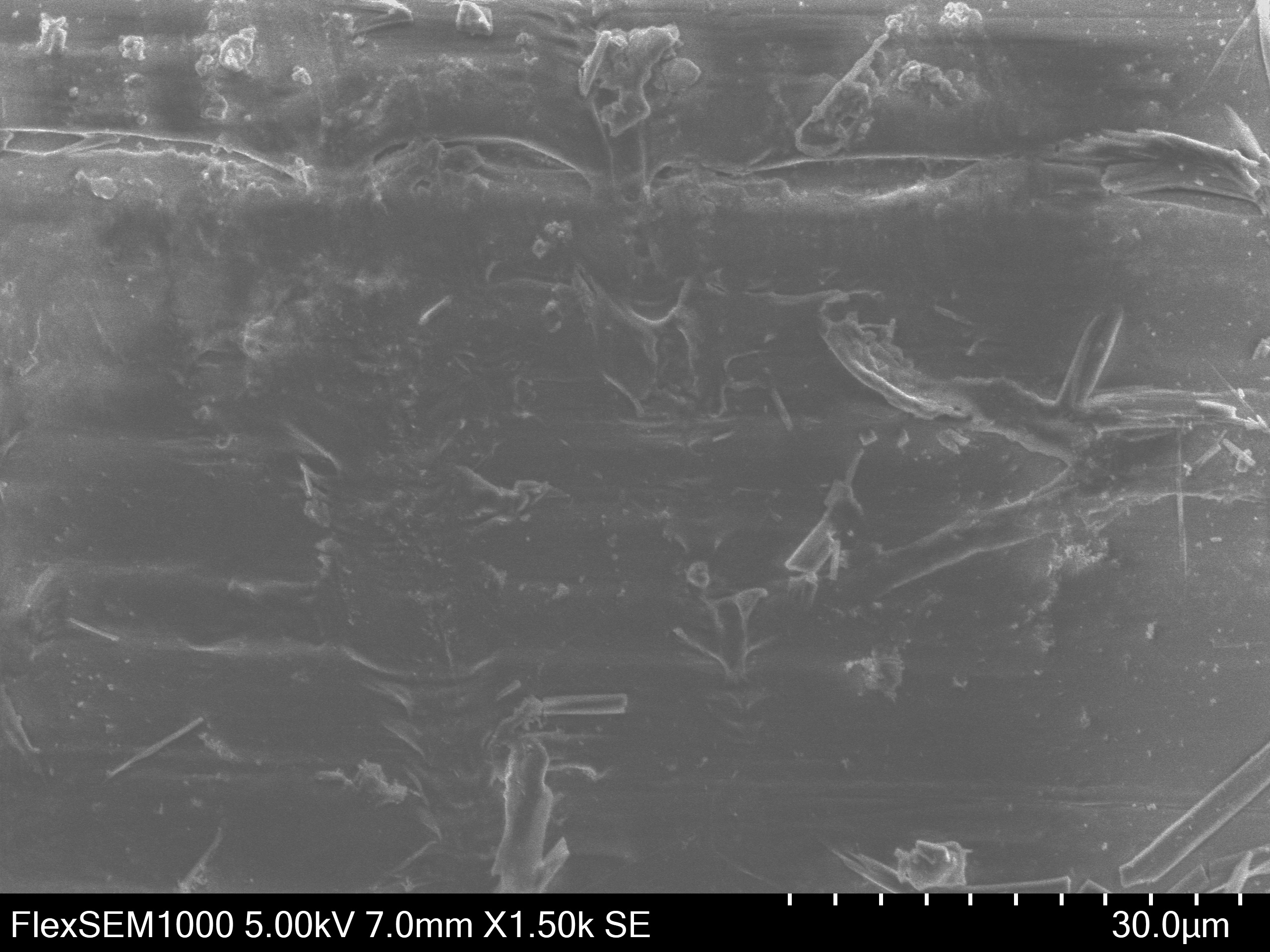

Supplement: Supplementary file 1 [file materials-14-05462-s001.zip › Gallery S1 SEM images of stent surface cracks/4weeks_02_x1500_SE.jpg]

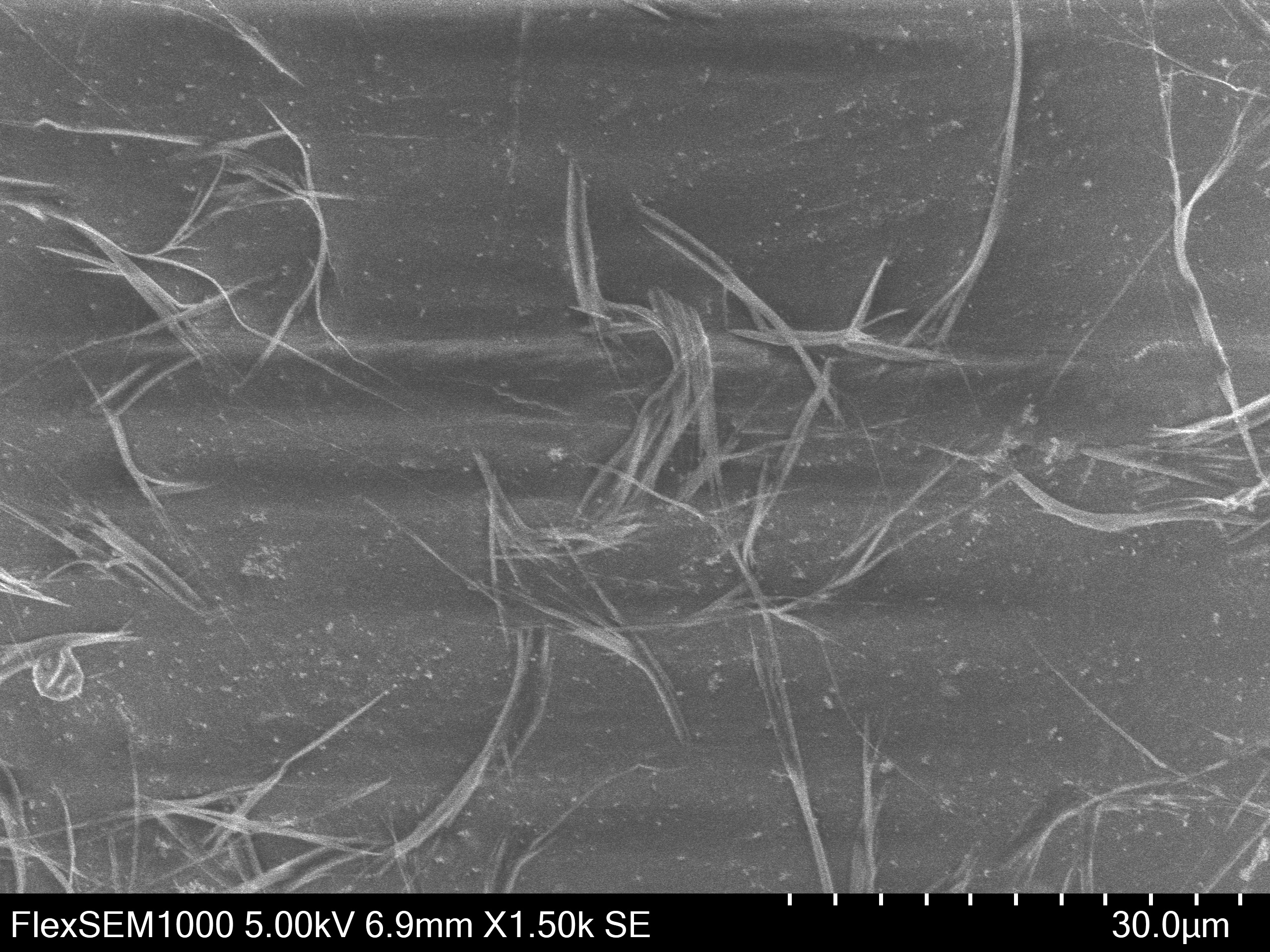

Supplement: Supplementary file 1 [file materials-14-05462-s001.zip › Gallery S1 SEM images of stent surface cracks/4weeks_03_x1500_SE.jpg]

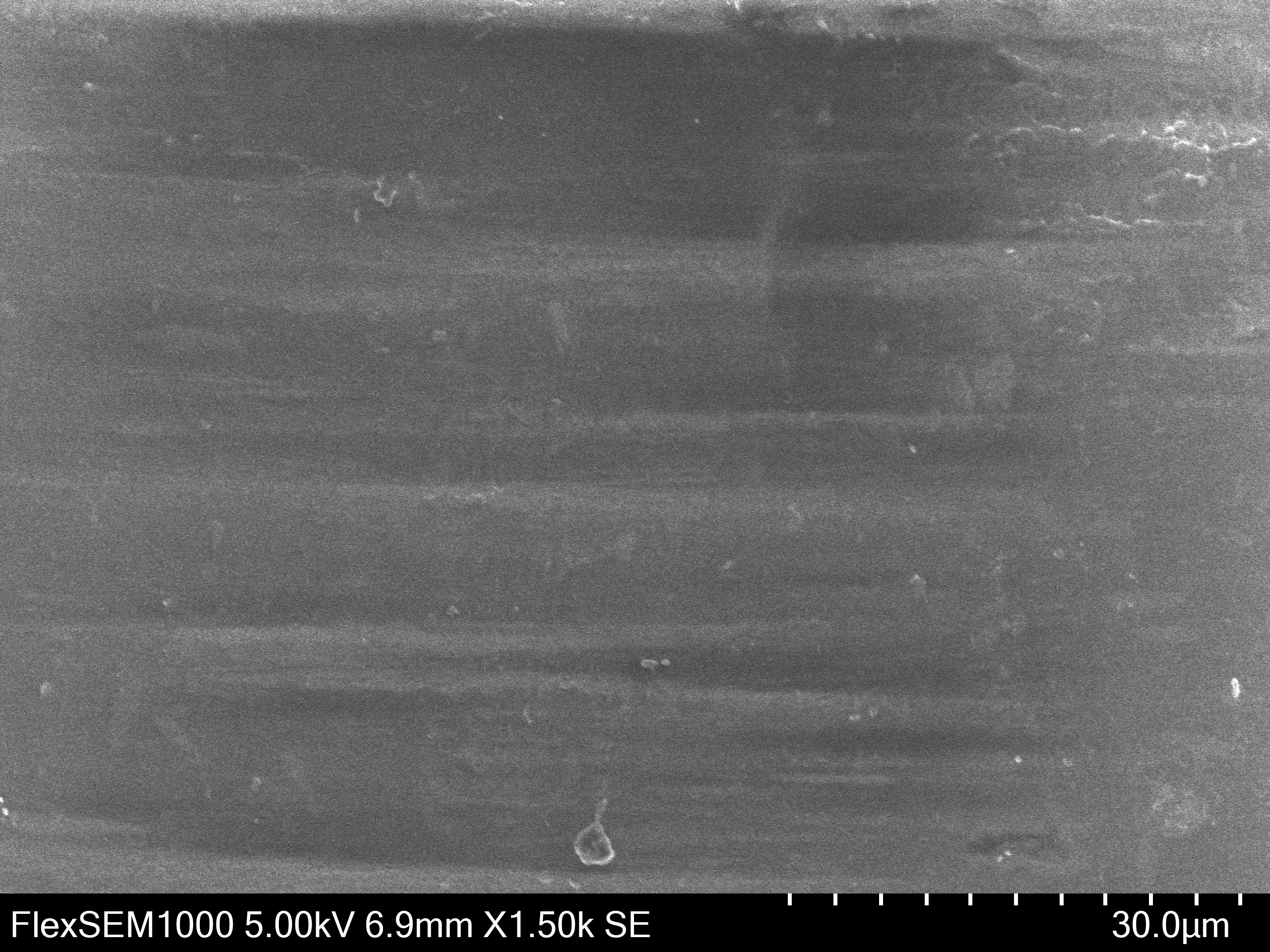

Supplement: Supplementary file 1 [file materials-14-05462-s001.zip › Gallery S1 SEM images of stent surface cracks/4weeks_04_x1500_SE.jpg]

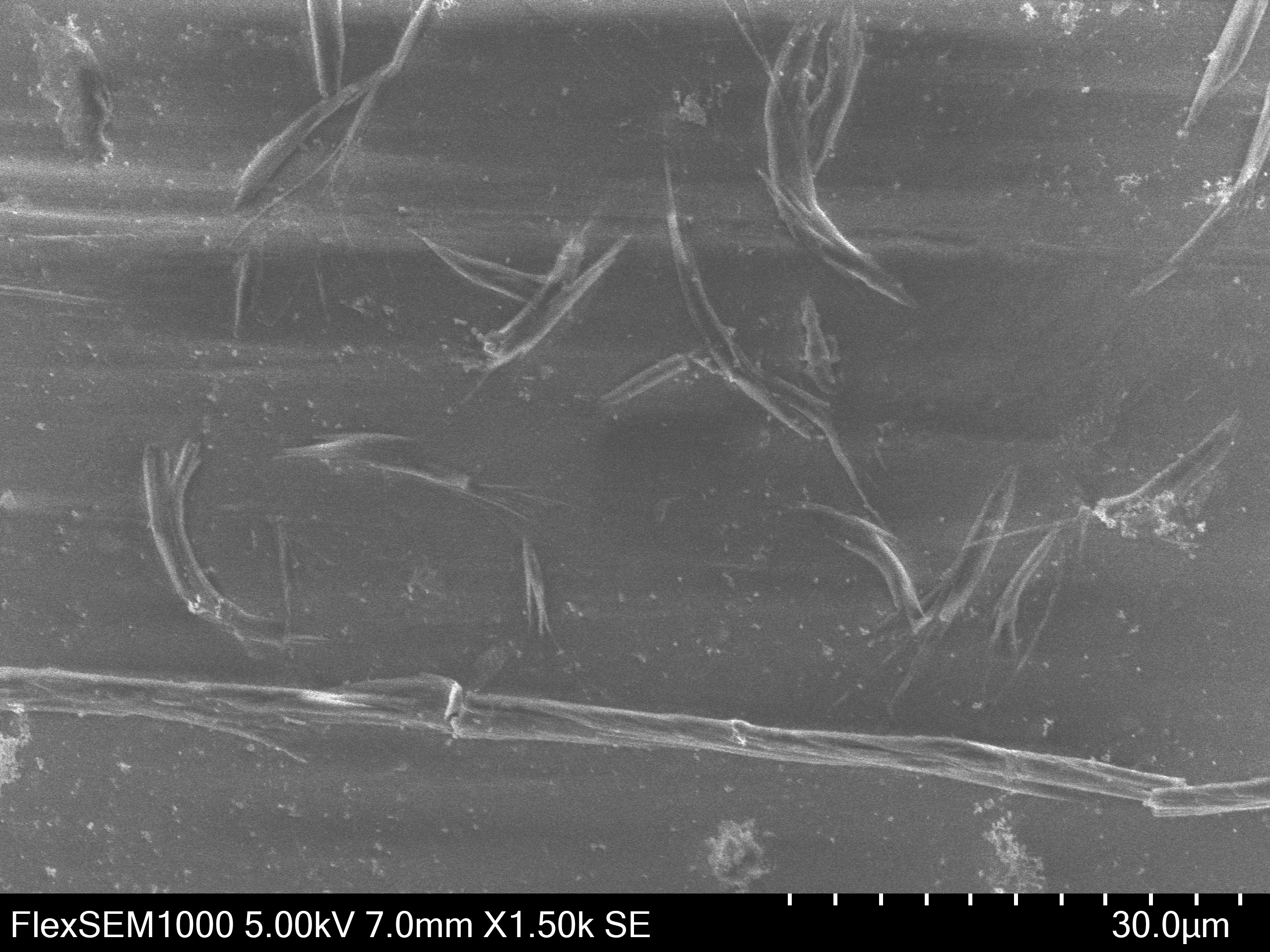

Supplement: Supplementary file 1 [file materials-14-05462-s001.zip › Gallery S1 SEM images of stent surface cracks/4weeks_05_x1500_SE.jpg]

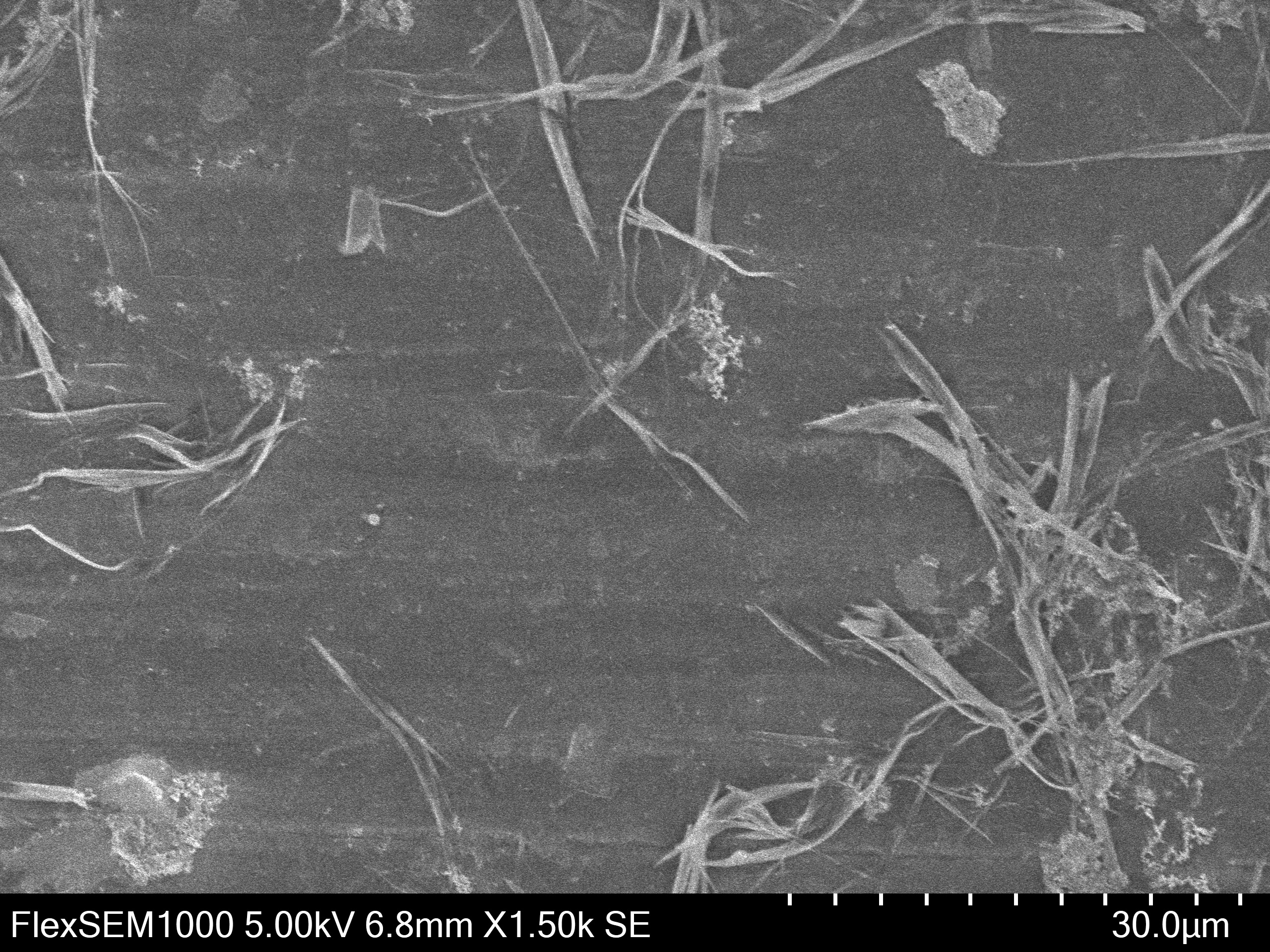

Supplement: Supplementary file 1 [file materials-14-05462-s001.zip › Gallery S1 SEM images of stent surface cracks/8weeks_01_x1500_SE.jpg]

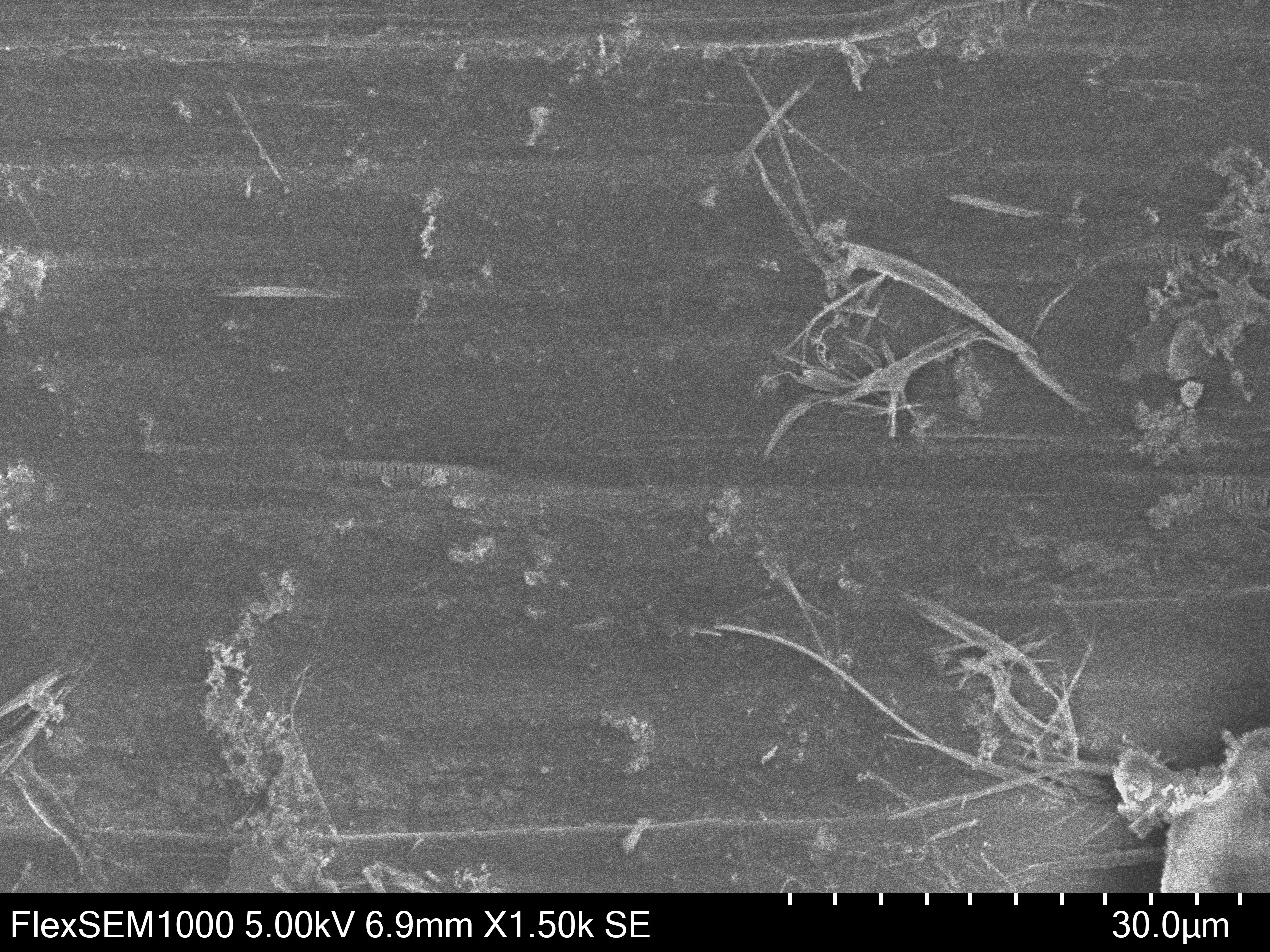

Supplement: Supplementary file 1 [file materials-14-05462-s001.zip › Gallery S1 SEM images of stent surface cracks/8weeks_02_x1500_SE.jpg]

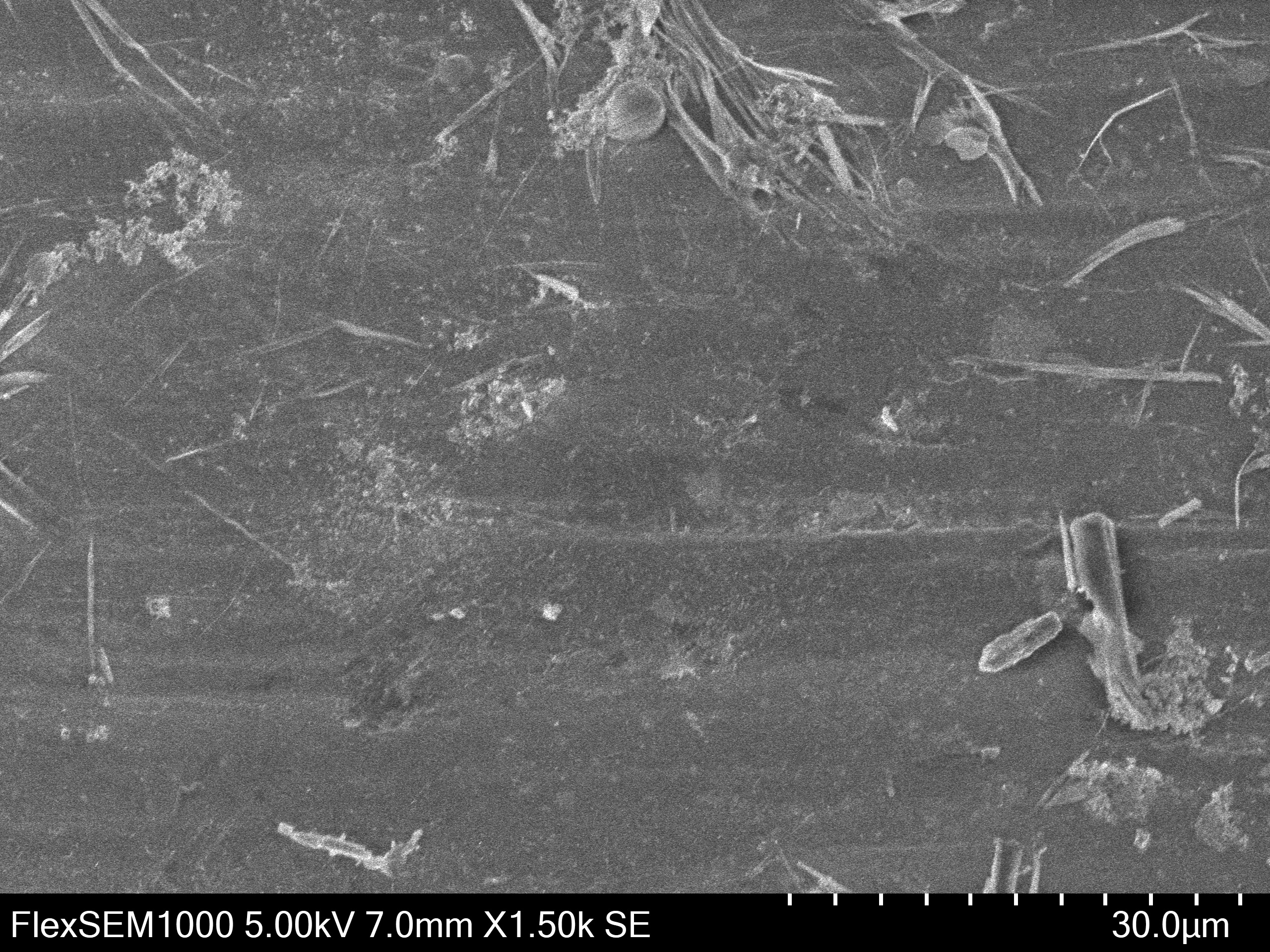

Supplement: Supplementary file 1 [file materials-14-05462-s001.zip › Gallery S1 SEM images of stent surface cracks/8weeks_03_x1500_SE.jpg]

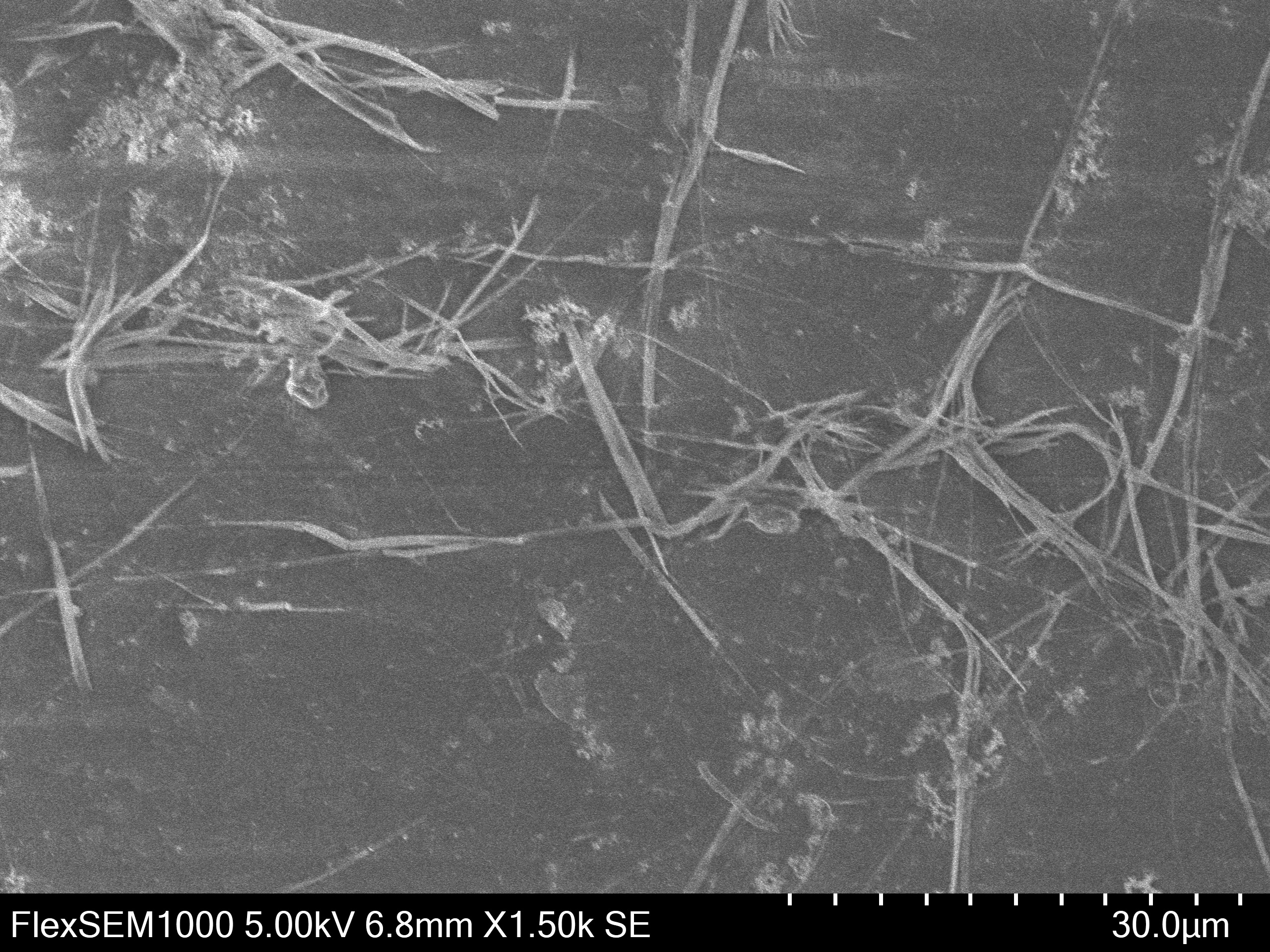

Supplement: Supplementary file 1 [file materials-14-05462-s001.zip › Gallery S1 SEM images of stent surface cracks/8weeks_04_x1500_SE.jpg]

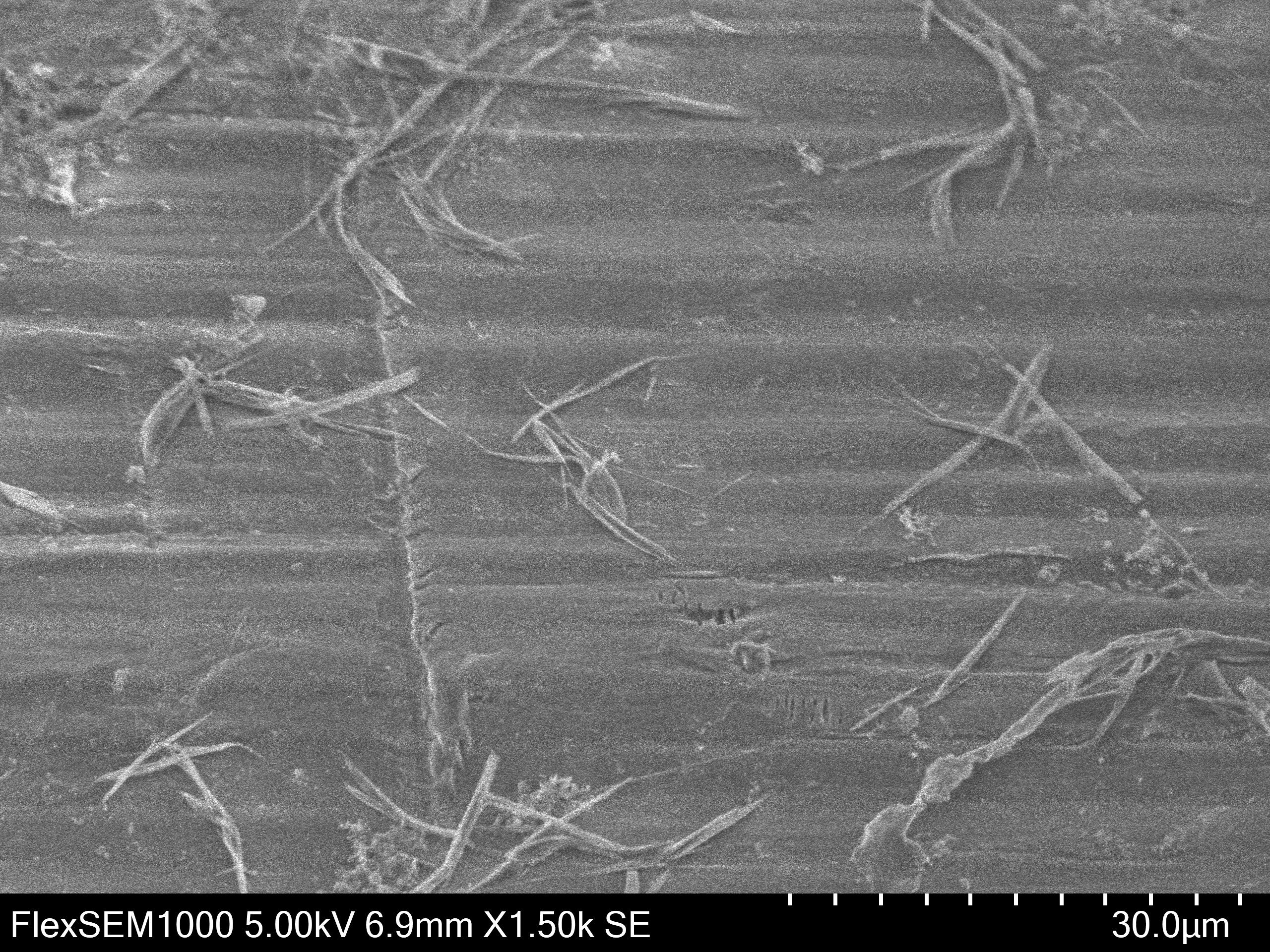

Supplement: Supplementary file 1 [file materials-14-05462-s001.zip › Gallery S1 SEM images of stent surface cracks/8weeks_05_x1500_SE.jpg]

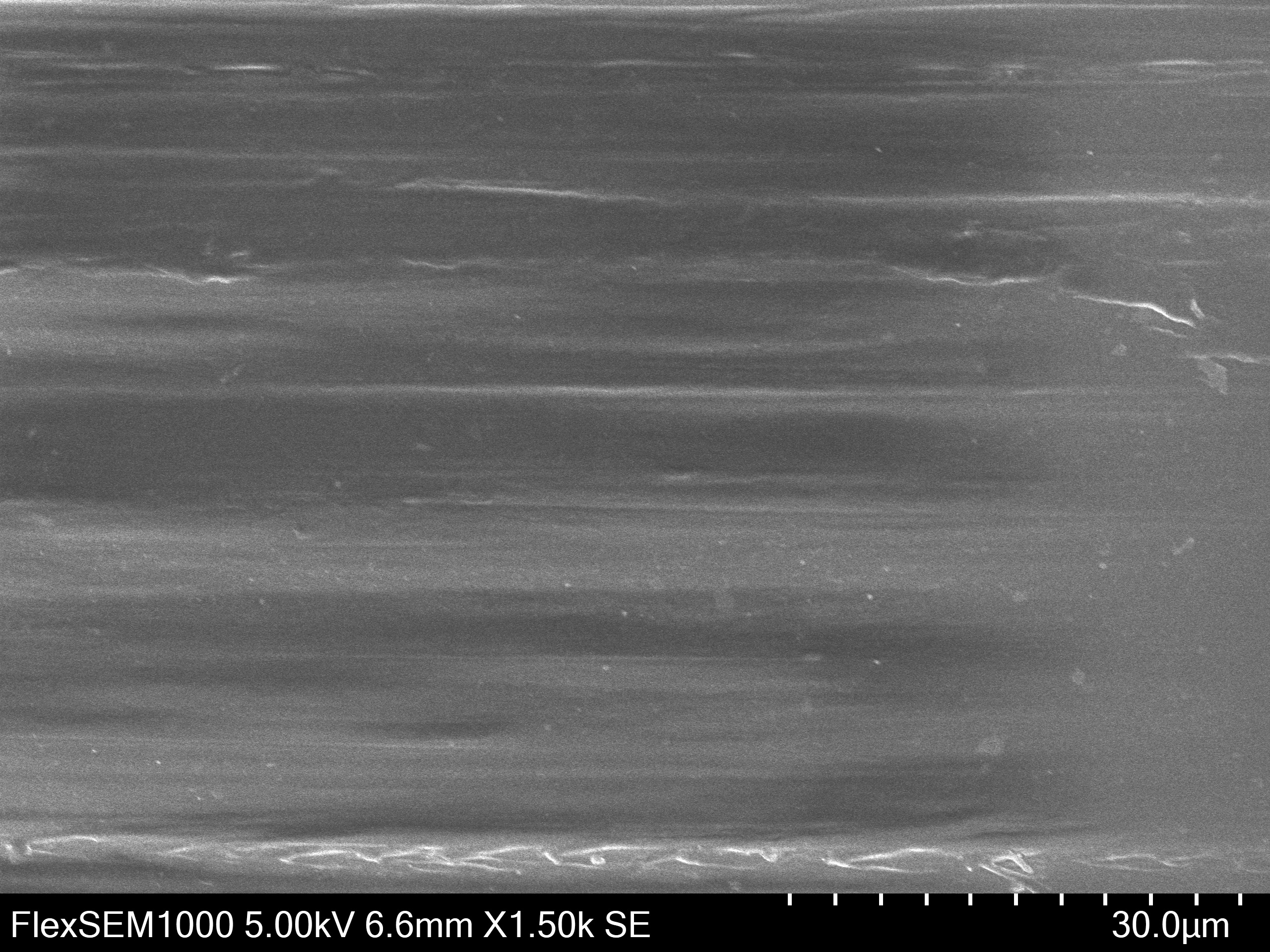

Supplement: Supplementary file 1 [file materials-14-05462-s001.zip › Gallery S1 SEM images of stent surface cracks/non-degraded_01_x1500_SE.jpg]

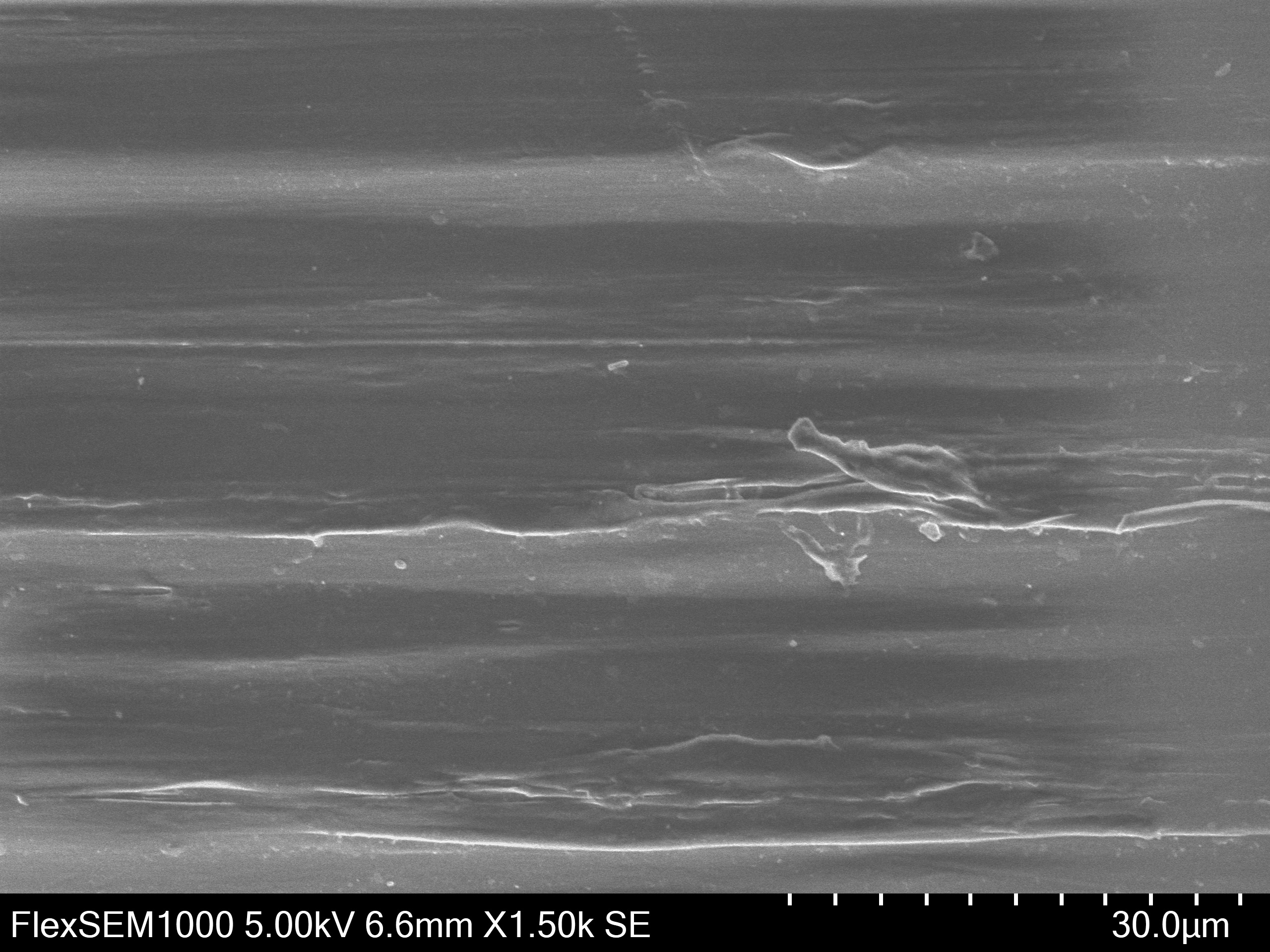

Supplement: Supplementary file 1 [file materials-14-05462-s001.zip › Gallery S1 SEM images of stent surface cracks/non-degraded_02_x1500_SE.jpg]

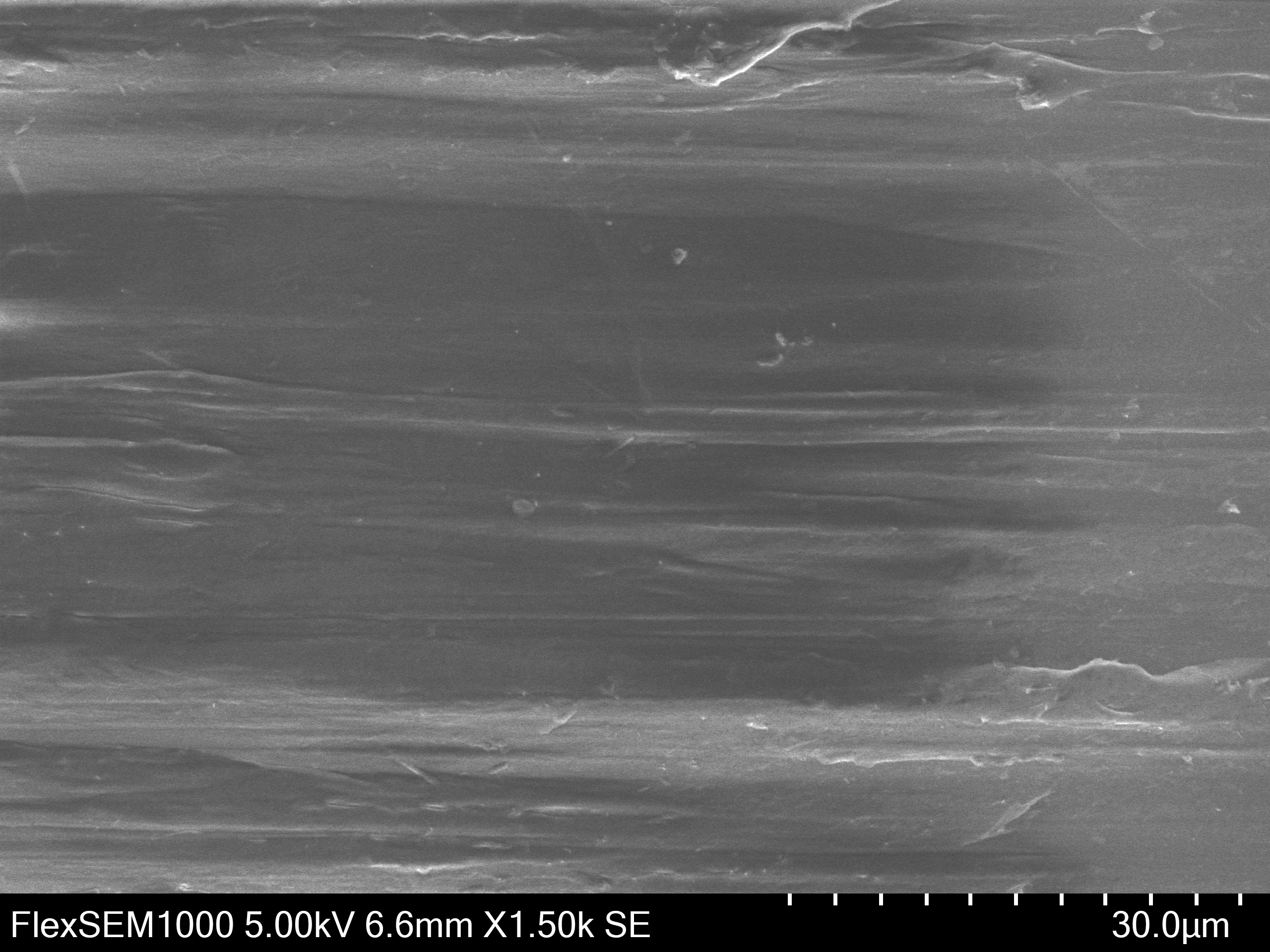

Supplement: Supplementary file 1 [file materials-14-05462-s001.zip › Gallery S1 SEM images of stent surface cracks/non-degraded_03_x1500_SE.jpg]

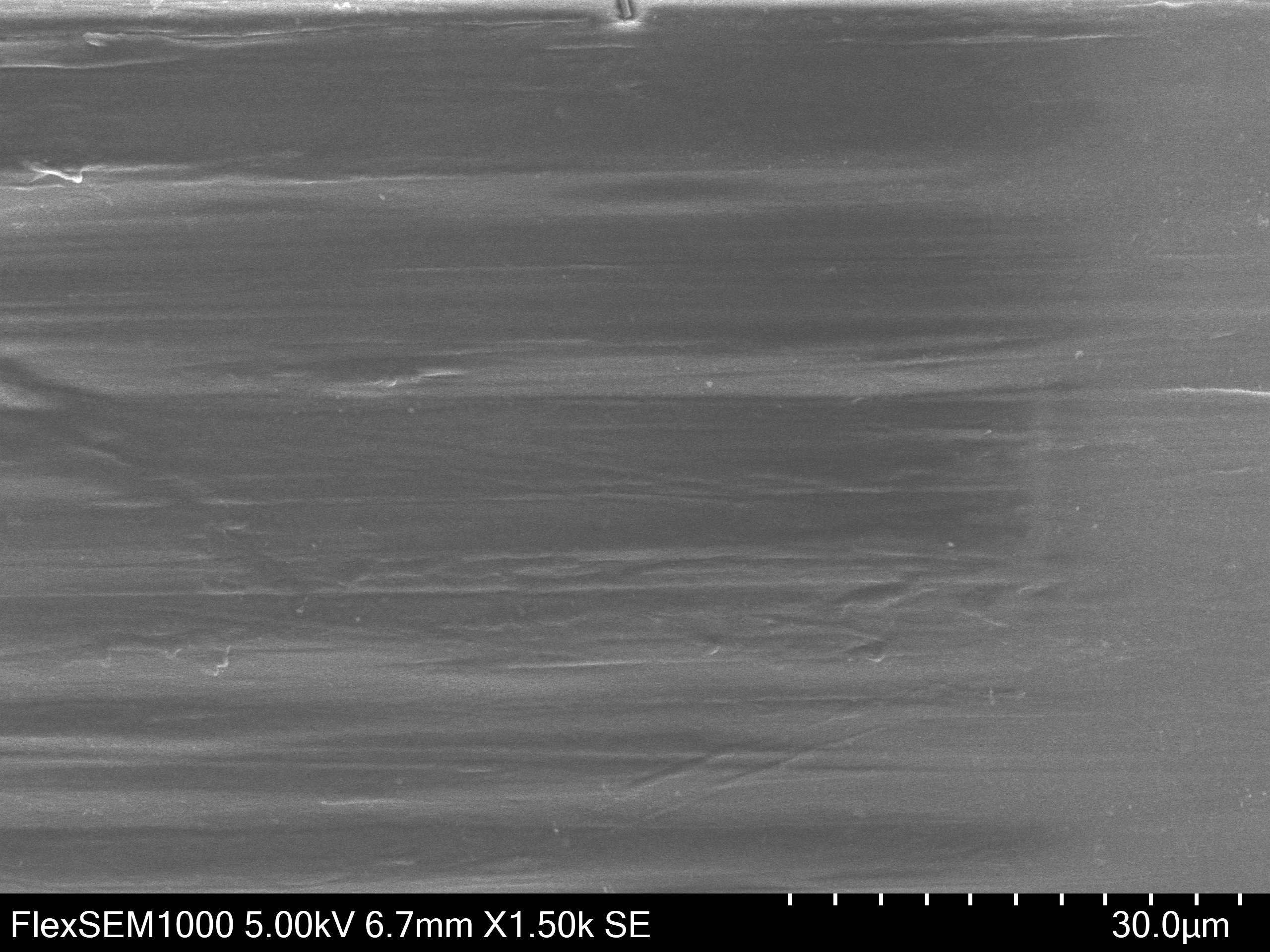

Supplement: Supplementary file 1 [file materials-14-05462-s001.zip › Gallery S1 SEM images of stent surface cracks/non-degraded_04_x1500_SE.jpg]

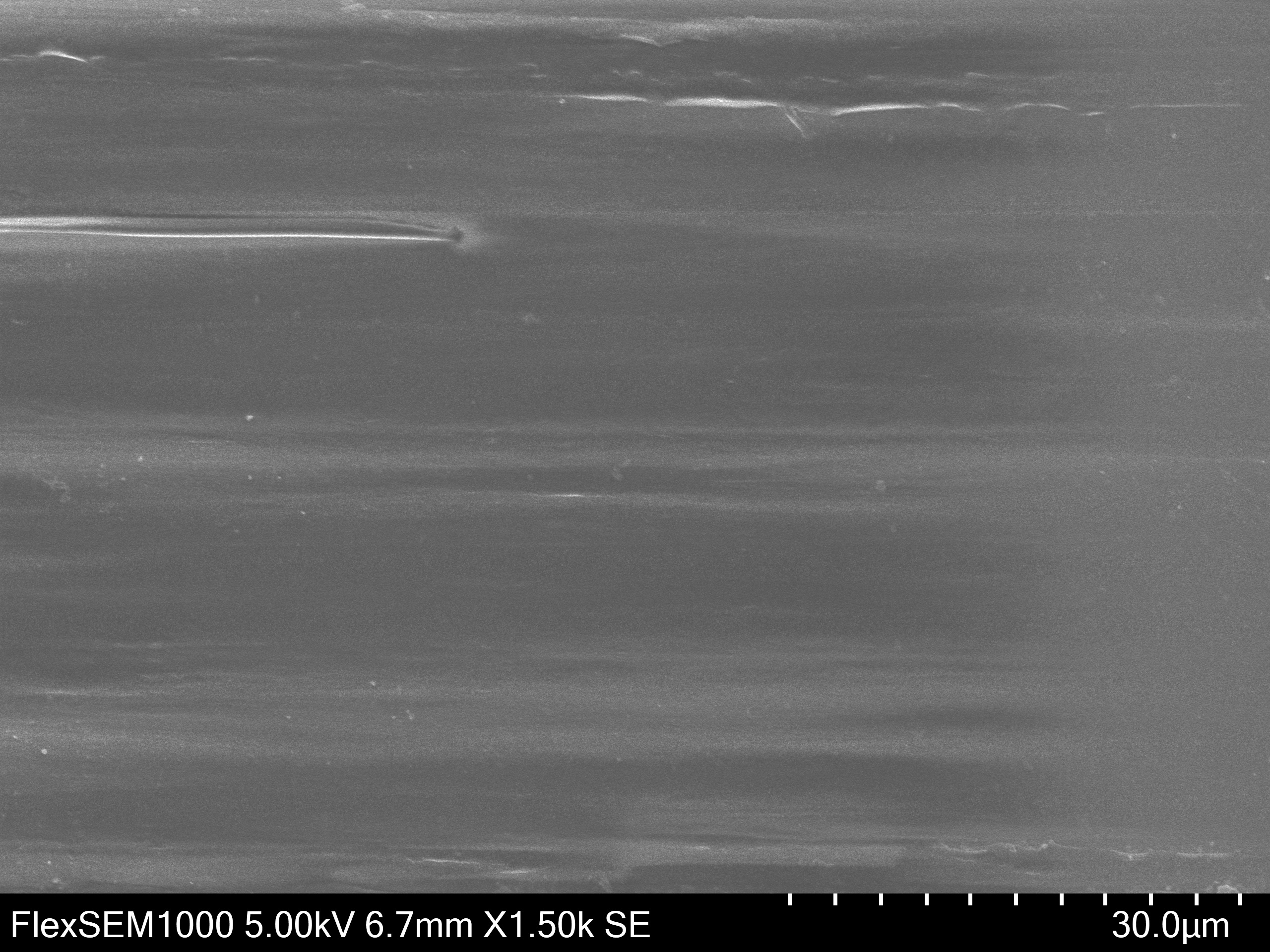

Supplement: Supplementary file 1 [file materials-14-05462-s001.zip › Gallery S1 SEM images of stent surface cracks/non-degraded_05_x1500_SE.jpg]
